# Preclinical evaluation of patient-specific 3D-printed PDLLA/SrHAp implants in a minipig segmental mandibular defect model

**DOI:** 10.1016/j.mtbio.2026.103523

**Published:** 2026-08-01

**Authors:** Christian Bräuer, Elisabeth Jarmer, Anja Lode, Tatjana Fecht, Stefanie Grom, Andreas Hoess, Richard Frank Richter, Martin Scharffenberg, Jakob Wittenstein, Roland Jung, Sascha Heinemann, Tobias Wolfram, Frank Reinauer, Michael Gelinsky, Günter Lauer, Corina Vater

**Affiliations:** a Department of Oral & Maxillofacial Surgery, University Hospital Carl Gustav Carus at Technische Universität Dresden, Fetscherstraße 74, Dresden, 01307, Germany; b Centre for Translational Bone, Joint and Soft Tissue Research, University Hospital Carl Gustav Carus and Faculty of Medicine at Technische Universität Dresden, Fetscherstraße 74, Dresden, 01307, Germany; c KLS Martin SE & Co. KG, Kolbinger Str. 10, Mühlheim, 78570, Germany; d INNOTERE GmbH, Meissner Str. 191, Radebeul, 01445, Germany; e Department of Anesthesiology and Intensive Care Medicine, Pulmonary Engineering Group, University Hospital Carl Gustav Carus at Technische Universität Dresden, Fetscherstraße 74, Dresden, 01307, Germany; f Experimental Center of the Faculty of Medicine, University Hospital Carl Gustav Carus and Faculty of Medicine at Technische Universität Dresden, Fetscherstraße 74, Dresden, 01307, Germany

**Keywords:** Patient-specific implants, Poly(DL-Lactide) (PDLLA), Calcium phosphate, Additive manufacturing, Bone, *In vivo*

## Abstract

Large segmental mandibular defects remain difficult to reconstruct, and patient-specific biodegradable scaffolds produced by additive manufacturing may offer an alternative to permanent alloplastic implants. This exploratory study evaluated three 3D-printed poly(DL-lactide) (PDLLA)-based implant configurations in a periosteum-preserving minipig mandibular defect model: PDLLA, PDLLA containing 10 wt% strontium-modified hydroxyapatite microparticles (PDLLA + SrHAp-P), and a biphasic PDLLA-shell/SrHAp-core implant (PDLLA/SrHAp-C). Twelve adult minipigs received patient-specific implants and titanium reconstruction plates (n = 4 per group). Bone formation was monitored by digital volume tomography and sequential fluorochrome labeling for 6 months; one animal per group underwent non-terminal follow-up to 14 months.

Complications occurred in 11 of 12 animals and included dehiscence, abscess or fistula formation, implant swelling, screw loosening, and fracture or displacement of the SrHAp core. Bone bridging was radiographically observed in all animals by 6 months, including an animal in which the implant had been removed prematurely, indicating a major contribution of the preserved periosteum and host tissues. New bone formed mainly around, rather than within, the implants. Implant-associated bone ingrowth was observed only in the biphasic group, but was not a uniform finding and fibrous encapsulation was frequent. The 3 animals followed to 14 months showed continued consolidation without terminal sacrifice.

These findings identify degradation-associated tissue reactions, dimensional instability, soft-tissue coverage, and construct mechanics as major limitations of large PDLLA-based mandibular implants. The biphasic architecture showed a potentially favorable local osteointegration pattern, but the small sample size and high complication rate preclude claims of efficacy or superiority. Further optimization of polymer content, ceramic architecture, porosity, mechanical stability, and soft-tissue management is required before additional translational studies.

## Highlights

• Challenging mandibular defect model with high infection risk.
• SrHAp integrated into PDLLA as microparticles or as a biphasic shell/core design.
• Defect bridging by 3D-printed patient-specific PDLLA-based implants by 6 months.
• Implant-associated bone ingrowth was observed only in the biphasic group.
• Non-terminal 14-month follow-up of one animal per group supported the 3 R principle.

## Graphical abstract

**Figure:**
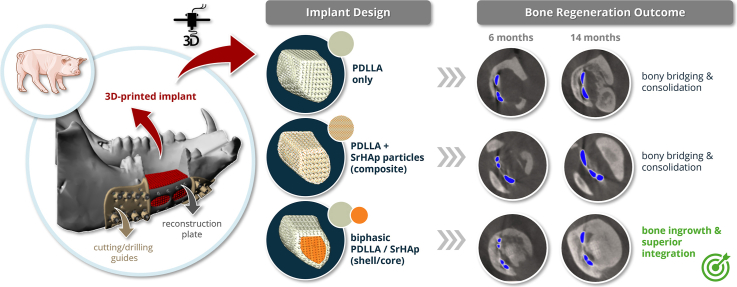

## 1 Introduction

Essential functions of the human body such as chewing, swallowing and breathing, as well as speech and facial expression, depend crucially on the integrity of the mandible. Any injury to this anatomical structure therefore causes substantial problems; in addition to impaired physiological functions, hindered communication and aesthetic disfigurement can lead to social isolation of the patient [1]. Diseases such as malignancies of the oral cavity, osteoradionecrosis or medication-related osteonecrosis of the jaw (MRONJ) can require subtotal mandibulectomy. Other conditions such as benign tumors, extensive cystic lesions, osteomyelitis and trauma can also lead to massive bone defects [2]. Adequate reconstruction of mandibular continuity defects is essential for the functional and aesthetic rehabilitation of patients, but is complex due to the multiple purposes of the mandible which include ensuring the aforementioned functions and shaping the face [3].

The use of autologous microvascular bone grafts is the current gold standard for mandibular reconstruction [3,4]. However, its principal disadvantage is donor-site morbidity associated with bone harvesting from the iliac crest, scapula or fibula [5]. Elderly patients with multiple morbidities may have difficulty compensating for this, which can lead to permanent immobility; limitations in physical performance and aesthetic impairments may also persist in younger patients [6,7]. In addition, the required grafts are not always available because of peripheral vascular compromise, the presence of osseous pathology at the donor sites, or the lack of anastomotic options. Furthermore, the amount of available bone is limited and the shape of the harvested bone does not exactly match the defect; complex shapes are difficult to achieve [5,8].

If sufficient autologous bone graft is not available, alternative reconstruction approaches are used in clinical practice, although they are associated with high complication rates. Alloplastic reconstruction using a titanium reconstruction plate alone [9] often leads to fracture of the plate or its exposure due to soft tissue shrinkage resulting in mucosal dehiscence, which in turn can lead to infection [10,11]. Encasement of the reconstruction plate with the thermoplastic polymer polymethyl methacrylate (PMMA) in the resection area counteracts soft tissue dehiscence – this approach is suitable for creating a temporary placeholder prior to secondary bone reconstruction and offers a definitive option in palliative situations [[12], [13], [14], [15], [16]]. Implants made of polyetheretherketone (PEEK), a thermoplastic polymer that, like PMMA, is non-degradable and bioinert but possesses favorable mechanical properties, have been described to be suitable for moderate-load, aesthetically sensitive regions such as the mandibular angle; their complication rates are comparable to those of titanium implants [17]. However, these approaches do not provide true mandibular regeneration, as the materials are not biodegradable, do not enable or even support new bone formation in the defect area and do not allow for dental rehabilitation.

The ideal biomaterial-based implant for mandibular reconstruction leads to complete bone regeneration and enables an anatomically correct restoration. The biomaterial(s) must therefore be resorbable, osteoconductive and ideally osteoinductive. To ensure undisturbed healing, the biomaterial or material combination must also remain biocompatible during degradation and in contact with the surrounding soft tissue. The suitability of a biomaterial for processing by additive manufacturing (AM) methods opens up the possibility of producing patient-specific implants even for complex-shaped defects. In addition, AM enables the creation of a porous structure according to a predefined design that facilitates tissue ingrowth and vascularization. The process chain for CAD/CAM (computer-aided design and manufacturing) fabrication of implants based on clinical imaging data (computed tomography (CT) or magnetic resonance imaging (MRI)) is established [18]. Currently, research is focusing on the development of biomaterials that meet the above-mentioned criteria and can be processed using AM methods such as 3D printing approaches.

Biomaterials based on poly(lactic acid) (PLA) are promising candidates. They are biodegradable, biocompatible, possess favorable mechanical properties and have already been extensively used in orthopaedic and dental applications – so far only as fixation devices such as screws, pins, washers, and bolts or membranes for bone augmentation [19,20]. The degradation kinetics of the material can be adjusted by the choice of the isoforms: Poly(L-lactide) (PLLA) and poly(D-lactide) (PDLA) exhibit longer degradation times due to their (semi)crystallinity than the amorphous poly(DL-lactide) (PDLLA) [[19], [20], [21]]. The degradation can also be accelerated by co-polymerization with other monomers, resulting in copolymers such as poly(lactic acid-co-glycolic acid) [19]. As thermoplastics, PLA derivatives are well suited for processing by 3D printing techniques such as fused filament fabrication [22] and Arburg Plastic Freeforming [23,24]. Arburg Plastic Freeforming is a nozzle-based additive manufacturing process in which thermoplastic polymer pellets are plasticized and dispensed as discrete droplets by a high-frequency pulsed nozzle. Computer-controlled layer-wise deposition of these droplets permits the fabrication of individualized porous constructs without the need to produce a filament feedstock [25].

One major disadvantage of PLA derivatives in large implants is the formation of acidic degradation products. The decrease of the pH value in the surrounding microenvironment can lead to inflammatory processes and damage cells involved in tissue regeneration [26]. However, integrating micro- and nanoparticles of basic mineral phases can reduce the acidification [23,27,28]. We have recently developed composite materials in which various mineral phases are integrated into a PDLLA matrix as microparticle fillers and which can be processed using the Arburg Plastic Freeforming method [24]. Degradation analysis of 3D-printed scaffolds over 24 weeks showed that CaCO_3_ was most effective in counteracting acidification, but led to a very strong increase in scaffold dimensions *in vitro* and *in vivo*, rendering PDLLA + CaCO_3_ composites unsuitable as an implant material for mandibular reconstruction. In contrast, calcium phosphate (CaP) phases (α/β-tricalcium phosphate, hydroxyapatite) also exhibited significant buffering capacity, but only caused moderate swelling of the respective composite scaffolds [24].

An alternative approach for combining thermoplastic polymers with a CaP phase is the generation of biphasic implants, in which the two materials are not mixed but are in close proximity to each other. In this configuration, the polymer and CaP phases are extruded separately, as we recently demonstrated for poly(L-lactic-co-glycolic) acid (PLLA-PGA) and a strontium-modified calcium phosphate cement (SrCPC) paste [29,30], which were deposited alternately in strands using a multi-tool 3D printing system [31]. While the dimensional changes observed for the composite materials [23,24] could be avoided with this strategy, the CaP phase in the SrCPC strands was still able to buffer the acidic degradation products released from the polymer strands in this biphasic configuration.

**Fig. 1:**
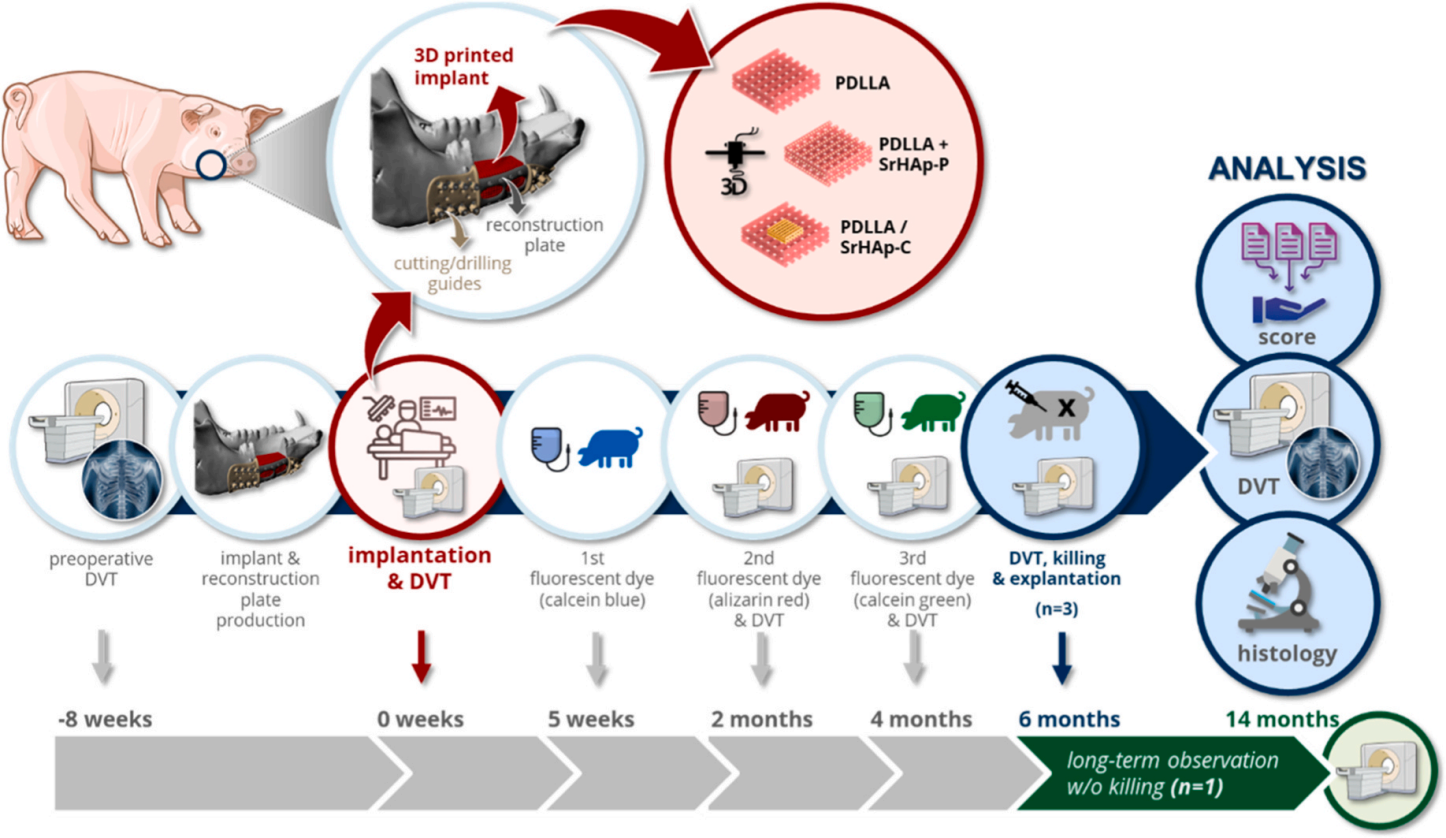
Study design illustrating the defect model, implant variants, timeline, interventions, and analytical methods (PDLLA: poly(DL-lactide), SrHAp: strontium-containing hydroxyapatite, DVT: digital volume tomography).

**Fig. 2:**
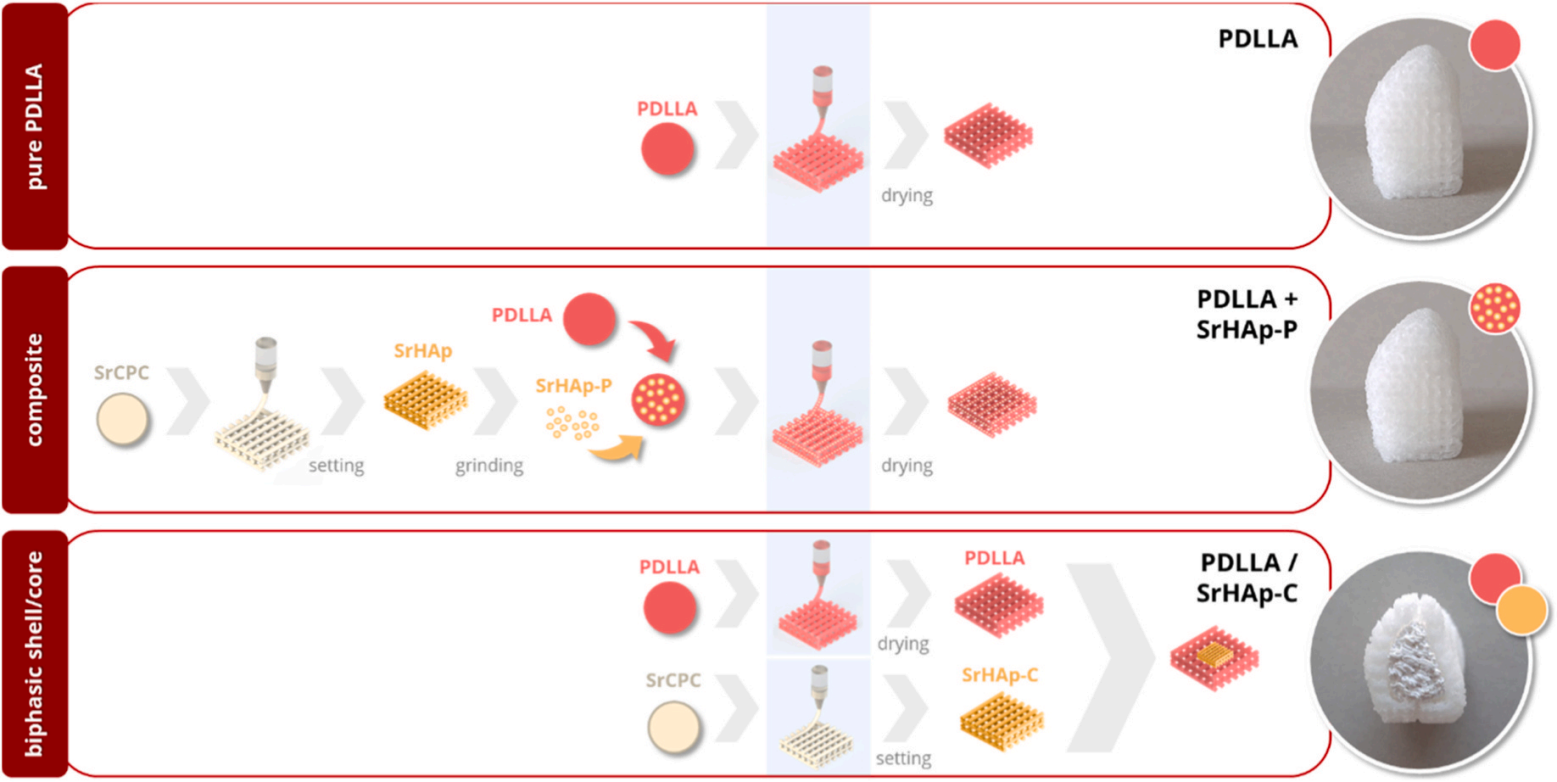
Production process of the implants used in the study (PDLLA: poly(DL-lactide), SrCPC: strontium-containing calcium phosphate cement, SrHAp: strontium-containing hydroxyapatite).

The overall objective of the present study was to evaluate the applicability of patient-specific 3D-printed, degradable implants consisting of a combination of PLA and CaP phases for mandibular reconstruction in a clinically relevant *in vivo* animal defect model (Fig. 1). Based on our previous study [24], we chose PDLLA (approved by FDA (U.S. Food and Drug Administration) K080862) as PLA derivative, and strontium-containing hydroxyapatite (SrHAp) as the CaP phase. SrHAp is formed during setting of the SrCPC and has been shown to be osteoconductive and resorbable and to release strontium ions at concentrations that support osteogenesis [29,32,33]. The two materials were either combined by mixing set SrHAp microparticles (SrHAp-P) into the PDLLA matrix to form an extrudable composite material (PDLLA + SrHAp-P), or PDLLA and SrCPC paste were extruded separately and after setting of the cement part, biphasic PDLLA/SrHAp-C implants with a shell/core structure were built (Fig. 2). A segmental mandibular defect model was established in minipigs (Fig. 1). Individual implants, which precisely fit into these defects, were designed and 3D-printed on the basis of CT data. After implantation into the defect and stabilization with a reconstruction plate, the performance of the composite and the biphasic implants was analyzed in comparison to pure PDLLA implants with regard to bone defect regeneration over an observation period of 6 months. In order to more closely approximate the clinical situation, 1 animal per group underwent long-term observation, involving CT examination, biopsy, and removal of the reconstruction plate after 14 months.

## 2 Materials and methods

### 2.1 Study design

Twelve adult minipigs were randomly allocated into three groups: 1) PDLLA pure polymer implants, 2) PDLLA + SrHAp-P composite implants, and 3) PDLLA/SrHAp-C biphasic (shell/core) implants (n = 4 per group). The unit of analysis was the individual animal. Allocation procedures (block randomization) and efforts to minimize bias were implemented.

In each minipig, a right-sided segmental mandibular defect was created between the first premolar and the last molar. The defect was filled with an individually manufactured implant and stabilized using a patient-specific titanium reconstruction plate. To monitor the progress of bone regeneration over an observation period of 6 months, fluorescent dyes were administered at 5 weeks and at 2 and 4 months after implantation and digital volume tomography (DVT) was carried out 0, 2, 4, and 6 months after implantation. Three animals of each group were killed after 6 months and the explanted specimens were analyzed macroscopically, radiographically, and histologically. Blinding of outcome assessment was not feasible in this study due to the inherently distinct structural and compositional characteristics of the implanted scaffolds. The biphasic PDLLA/SrHAp-C shell/core design and the composite PDLLA + SrHAp-P scaffolds exhibited identifiable features in DVT and histological sections, rendering group allocation apparent during analysis. In addition, the use of patient-specific titanium reconstruction plates and individualized surgical configurations further precluded anonymization of samples and imaging data. To mitigate potential bias, outcome measures were based on objective and quantitative assessments, including radiological evaluation of defect bridging and fluorochrome-based analysis of bone formation. Wherever possible, image analysis was performed using standardized and predefined criteria to ensure consistency and reproducibility.

One animal per group was followed up for 14 months to simulate the clinical situation: After X-ray confirmation of bone bridging, the reconstruction plate was removed and a bone biopsy was taken from the defect area and histologically analyzed. The study design is summarized in Fig. 1.

### 2.2 Animal experiment and ethics statement

Healthy, skeletally mature, castrated male Aachener minipigs (21.6 ± 1.2 months, 56.5 ± 6.5 kg) were purchased from Heinrichs Genetics GmbH (Waldfeucht, Germany). Prior to the experiments, they were housed in groups of four for a 10 day-acclimatization period. Under a 12-h light-dark cycle, the minipigs were fed twice daily with commercially available pellet food and given water *ad libitum*. After surgery, the food was mixed with water to facilitate food intake and minimize mechanical load. Adverse events, including soft tissue complications, infection, and implant instability, were recorded and are reported in detail in the Results section. This study was designed, conducted, and reported in accordance with the ARRIVE 2.0 guidelines developed by the NC3Rs [34]. All procedures were approved by the local animal-welfare authority of the District Government of Saxony, Dresden, Germany under protocol number 25-5131/496/46 and complied with national and international regulations governing animal research (German legislation for animal protection and the regulations for animal experiments of the state of Saxony, Directive 2010/63/EU).

### 2.3 DVT measurements

X-ray-based digital volume tomography (DVT) was carried out pre-operatively to provide anatomical data for the fabrication of individual implants as well as reconstruction plates and cutting/drilling guides. Postoperative DVT scans were performed immediately postoperatively and 2, 4, 6, and where applicable, 14 months to assess bone regeneration. Each animal underwent skull imaging under analgosedation, with supplemental oxygen provided by insufflation. Midazolam (1 mg/kg; Ratiopharm, Ulm, Germany), ketamine (10 mg/kg; Inresa Arzneimittel GmbH, Freiburg, Germany), and atropine (0.05 mg/kg; B. Braun, Melsungen, Germany) were administered intramuscularly. DVT was performed using the mobile xCAT scanner (Xoran, Ann Arbor, USA; 120 kV, 57.6 mAs, voxel size = 400 μm) and the data were analyzed using Dragonfly ORS (version 2021.1.0.977, Comet Technologies Canada Inc., Montreal, Canada) to create three-dimensional reconstructions and cross-sectional images from the sagittal, transverse, and coronal planes.

### 2.4 Design and fabrication of individual implants and reconstruction plates

Individual case planning as well as design and production of the implants and reconstruction plates were performed by KLS Martin SE & Co. KG (Mühlheim, Germany).

#### 2.4.1 Implant materials

PDLLA was obtained as pellets from Evonik Industries (Essen, Germany). SrCPC paste [29] and SrHAp microparticles, the latter obtained from set SrCPC (diameter < 50 μm), were provided by INNOTERE GmbH (Radebeul, Germany). The PDLLA + SrHAp-P composite material was prepared as described previously [24]. In brief, for melt compounding with PDLLA, the SrCPC was first fully set under humid conditions and converted into SrHAp, dried, milled and sieved to microparticles (diameter < 50 μm), since water-accessibility within the polymer matrix is limited and the hydraulic setting reaction is incompatible with thermoplastic melt processing. Then, the PDLLA powder and the SrHAp microparticles were mixed in a mass ratio of 90:10 using a Speed Mixer (Hauschild, Hamm, Germany), pressed into a block at temperatures of 150–200 °C, granulated in a cutting mill (Retsch, Haan, Germany) and dried under vacuum at 20 °C.

#### 2.4.2 Design and fabrication of the implants

The length of each implant corresponded to the planned defect length between the first premolar and the last molar. Depending on the anatomical dimensions of each animal, the final 3D-printed implants varied between 29.2 and 36.5 mm in length, 17.3 and 23.3 mm in width, and 22.1 and 27.6 mm in height (values for each animal are shown in the supplemental information, Supplemental Fig. A1). The cross-section of the implants corresponded to the cross-section of the bone stumps at the resection surfaces with a recess of approximately 0.5 cm cranially to enable subsequent tension-free suture adaptation and to compensate for possible swelling of the implants. Inside, the implants had a porous structure. Biphasic shell/core implants consisted of an outer shell made of PDLLA and an inner core made of SrHAp. Based on the external envelope and neglecting the internal porous architecture, mean implant-envelope volumes calculated using the software PrusaSlicer were 6.94 ± 1.06 cm^3^ for PDLLA, 6.16 ± 0.40 cm^3^ for the composite PDLLA + SrHAp-P, 3.76 ± 0.48 cm^3^ for the PDLLA shell of the biphasic PDLLA/SrHAp-C, and 5.35 ± 0.90 cm^3^ for the SrHAp core of the biphasic PDLLA/SrHAp-C (Supplemental Fig. A1).

Pure PDLLA implants and PDLLA + SrHAp-P composite implants were fabricated using the 3D printing system Freeformer 200-3X (Arburg, Lossburg, Germany) as described previously [24]. In brief, pre-operative DVT data of the mandible were imported into the software Geomagic Freeform (Artec 3D, Senningerberg, Luxemburg), processed and the implants were designed individually, with a strand thickness of 1 mm and pore dimensions of 1.3 mm and 0.6 mm in x and y directions, respectively (Supplemental Fig. A2). After fabrication at an ambient chamber temperature of 40–60 °C (temperature inside the Freeformer) using a melt pressure of 30 MPa and a processing/printing temperature of 170–250 °C, the implants were vacuum dried for storage.

The co-printing of PDLLA and SrCPC in one process was not possible with the Freeformer printing system. Therefore, the biphasic shell/core implants – with a PDLLA shell and a SrHAp core – were designed and printed separately (Fig. 2). Using SrCPC, the core was extrusion-printed at 30 °C using a Bioplotter (Envisiontec, Gladbeck, Germany). After printing, the porous SrCPC core was incubated for 60 h at room temperature in a water-saturated humid atmosphere using deionized (DI) water to allow setting and conversion into SrHAp, washed in acetone and dried for 24 h at room temperature. The PDLLA shell had the same porous geometry as the PDLLA implants (2.4.2) and was fabricated in two halves using the Freeformer printing system as described above. After fabrication, the PDLLA shell was vacuum dried, assembled around the SrHAp core and fixed with SonicPins Rx® (KLS Martin SE & Co. KG).

The two configurations – the composite PDLLA + SrHAp-P and the biphasic PDLLA/SrHAp-C - therefore compared a dispersed dry SrHAp filler with a spatially separated, printed SrHAp phase.

#### 2.4.3 Design and fabrication of the cutting/drilling guides

The cutting/drilling guides were designed on the basis of the individual DVT data to match the shape of the bone surface, enabling the defined creation of the segmental defect between the first premolar and the last molar. They were printed from polyamide (PA) using the Freeformer printing system and vacuum dried for storage.

#### 2.4.4 Design and fabrication of the reconstruction plates

The patient-specific reconstruction plates were designed on the basis of the preoperative DVT data to match the individual mandibular anatomy of each animal. The plate design consisted of two horizontal struts that converged in the anterior submental region and were connected by three vertical struts. Two curved retention arms in the submandibular region secured the plate to the bone and helped maintain the position of the implant (Supplemental Fig. A3). The reconstruction plates were manufactured from Ti-6Al-4V titanium alloy according to ASTM F136 using selective laser melting on an SLM 125 system (NIKON SLM Solutions, Lübeck, Germany). A layer thickness of 20–50 μm was used during fabrication. After additive manufacturing, the plates underwent annealing, removal of the support structures, grinding, milling, abrasive blasting, and washing. Each plate was subsequently subjected to visual inspection, dimensional inspection, and functional testing before use.

#### 2.4.5 Sterilization

All implants were sterilized by gamma irradiation (low dose) at a validated absorbed dose of 15–30 kGy. The cutting/drilling guides, the screws for fixation and the reconstruction plates were steam-sterilized (121 °C for 20 min under vacuum conditions).

### 2.5 Surgical procedure for implantation

Prior to surgery, the minipigs received midazolam (1 mg/kg; Ratiopharm, Ulm, Germany), ketamine (10 mg/kg; Inresa Arzneimittel GmbH, Freiburg, Germany) and atropine (0.05 mg/kg; B. Braun, Melsungen, Germany) via intramuscular injection to minimize stress during transport to the operating room. For surgery, the minipigs were anesthetized using intravenous ketamine (15 mg/kg/h) and midazolam (1 mg/kg/h), orotracheally intubated, placed in supine position, and ventilated according to clinical recommendations for intra-operative lung-protective ventilation. Standard cardiorespiratory monitoring was established, and vital signs were monitored continuously. Fluid balance was maintained using intravenous balanced crystalloids (10 mL/kg/h). In addition, intraoperative analgesics (carprofen, 4 mg/kg; Rimadyl, Zoetis Deutschland GmbH, Berlin, Germany) as well as prophylactic antibiotics (amoxicillin, 15 mg/kg; Duphamox L.A., Zoetis Deutschland GmbH) were administered.

**Fig. 3:**
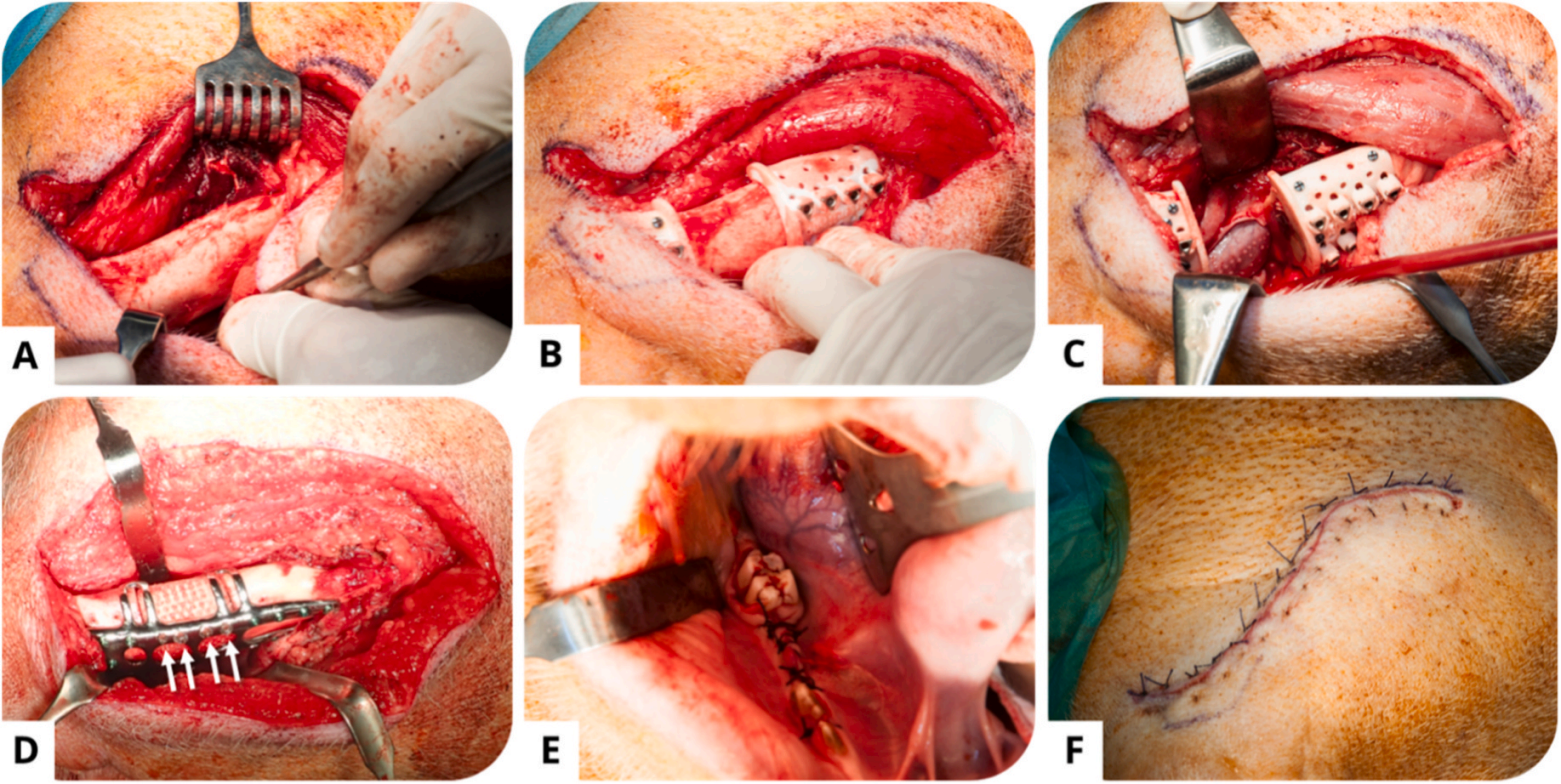
Surgical implantation procedure. (A) The soft tissue and periosteum were dissected to expose the mandible. (B) The patient-specific cutting and drilling guides were positioned, and holes for reconstruction-plate fixation were drilled. (C) The segmental osteotomy was performed. (D) The patient-specific implant and reconstruction plate were inserted, and the implant was fixed to the plate using SonicPins Rx® (white arrows). (E, F) The intraoral and extraoral incisions were closed in multiple layers.

Following anesthesia induction, the surgical site was disinfected and isolated with sterile drapes. An S-shaped skin incision was made a few centimeters inferior to the contour of the right mandible, below the jawbone, followed by soft tissue and periosteum dissection to expose the mandible. The cutting/drilling guides were temporarily fixed with screws (maxDrive®, Ø 2.0 mm, length: 7 mm; KLS Martin SE & Co. KG) to the bone and marked the correct position of the resection area and the position of the drill holes for the screws of the reconstruction plate. An oscillating bone saw (TRS Modular Drive with saw blade 70/49x20x0.6/0.4 mm, both: Synthes GmbH, Oberdorf, Switzerland) cooled with sterile saline solution was used to perform an osteotomy along the edges of the cutting guides to create the segmental defect. The individual reconstruction plate was fixed with titanium locking screws (maxDrive®, Ø 2.3/2.7 mm, length: 7–19 mm depending on the individual anatomical situation; KLS Martin SE & Co. KG) on the outer lateral side of the mandible (Supplemental Fig. A3). Then, the individual implant was inserted and fixed to the reconstruction plate with SonicPins Rx® using Sonic Weld technology (KLS Martin SE & Co. KG). The incisions were closed intra- and extraorally in a multilayer fashion using Vicryl Plus 0 (Ethicon, Norderstedt, Germany) for suturing. Finally, the skin wound was disinfected (Fig. 3).

After completion of the surgery and performance of the first postoperative DVT scan (0 months), spontaneous ventilation had resumed adequately and minipigs were extubated under stable cardiorespiratory conditions. The minipigs received transdermal fentanyl (plaster, 12 μg/h; Ratiopharm, Ulm, Germany) for 3 days and carprofen (4 mg/kg/d) or meloxicam (0.4 mg/kg/d; Metacam® suspension, Boehringer Ingelheim Vetmedica GmbH, Ingelheim/Rhein, Germany) orally for 10 days for postoperative pain management. Amoxicillin (1 tablet Amoclav, 875 mg Amoxicillin + 175 mg clavulanic acid per day; Hexal, Holzkirchen, Germany) was administered orally for 10 days to provide postoperative infection prophylaxis.

### 2.6 Interventions, killing, and long-term follow-up

To assess the healing process, to perform DVT scans, and/or to inject the dyes for sequential polychrome labeling, the minipigs received analgosedation at 5 weeks, 2, 4, and 6 months postoperatively via intramuscular injection of midazolam, ketamine and atropine as described above (see 2.3). For chronological visualization of new bone formation after explantation, the calcium-binding fluorescent dyes Calcein Blue (30 mg/kg body weight; Sigma-Aldrich, Cat.#M1255), Alizarin Complexone (30 mg/kg body weight; Honeywell Fluka, Cat.# 05590), and Calcein disodium salt (20 mg/kg body weight; Honeywell Fluka, Cat.# 21030) were dissolved in 2 % sodium bicarbonate, adjusted to pH 7.2, sterile-filtered and then infused slowly at approximately 1 drop/s via an ear vein after 5 weeks, 2, and 4 months, respectively.

At 6 months after implantation, 9 animals were killed under general anesthesia by intravenous bolus injection of 2 g thiopental (Inresa Arzneimittel GmbH, Freiburg, Germany) and 50 mL 1 M potassium chloride (Fresenius Kabi, Bad Homburg, Germany) in accordance with the ethical standards. The mandible was resected along with the surrounding tissue, examined and the operated area together with approximately 2 cm of the adjacent host bone was prepared for histologic evaluation.

For long-term observation, one animal per group was housed externally and returned to the experimental center after 14 months. Under analgosedation (as described above, see 2.3), DVT scans were performed. Once sufficient defect bridging had been confirmed by X-ray, the reconstruction plate was removed. Bone that had grown over the reconstruction plate was gently chiseled away. The wound area was then sutured in the same way as during implantation, and the animals received the same peri- and post-operative medication. After an observation period of at least 2 weeks, during which the chewing stability of the jaws was checked, the animals were transferred to private homes after a final veterinary check-up.

### 2.7 Analysis of new bone formation based on DVT data

To determine the amount of newly formed bone in the defect area, the Dragonfly ORS software (version 2021.1.0.977, Comet Technologies Canada Inc.) was used to define a specific volume of interest (VOI). The cylindrical VOI spanned the defect from the anterior to posterior resection margin and intentionally captured bone formed within and around the original implant envelope, including periosteal or exostotic bone (Supplemental Fig. A4). The radius was adjusted according to the individual anatomical conditions. The length of the cylinder was maintained throughout the 6-months period, with the position of the cylinder being oriented both to the still visible resection edges and to the screw holes of the reconstruction plate. Bone was defined as tissue with a density in the range of 200–705 mg/cm^3^. However, this density range produced false-negative results for specimens with bone confirmed by radiographic, optical, and clinical validation. To address these false negatives, we conducted a second measurement using an expanded density range of 100–705 mg/cm^3^ (Supplemental Fig. A4). Voxels with density values above 705 mg/cm^3^ were classified as osteosynthesis material. The plate and bone volumes (BV, cm^3^) within this VOI were calculated for all available DVT data from 0 to 14 months postoperatively. The immediate postoperative scan served as reference and was used to derive baseline values covering the volume of the implant itself and the number of artifacts caused by the reconstruction plate and screws. Thus, BV values presented in the results section are baseline-corrected values (baseline-corrected BV = BV at respective time point – BV at 0 months).

### 2.8 Histological analysis

**Fig. 4:**
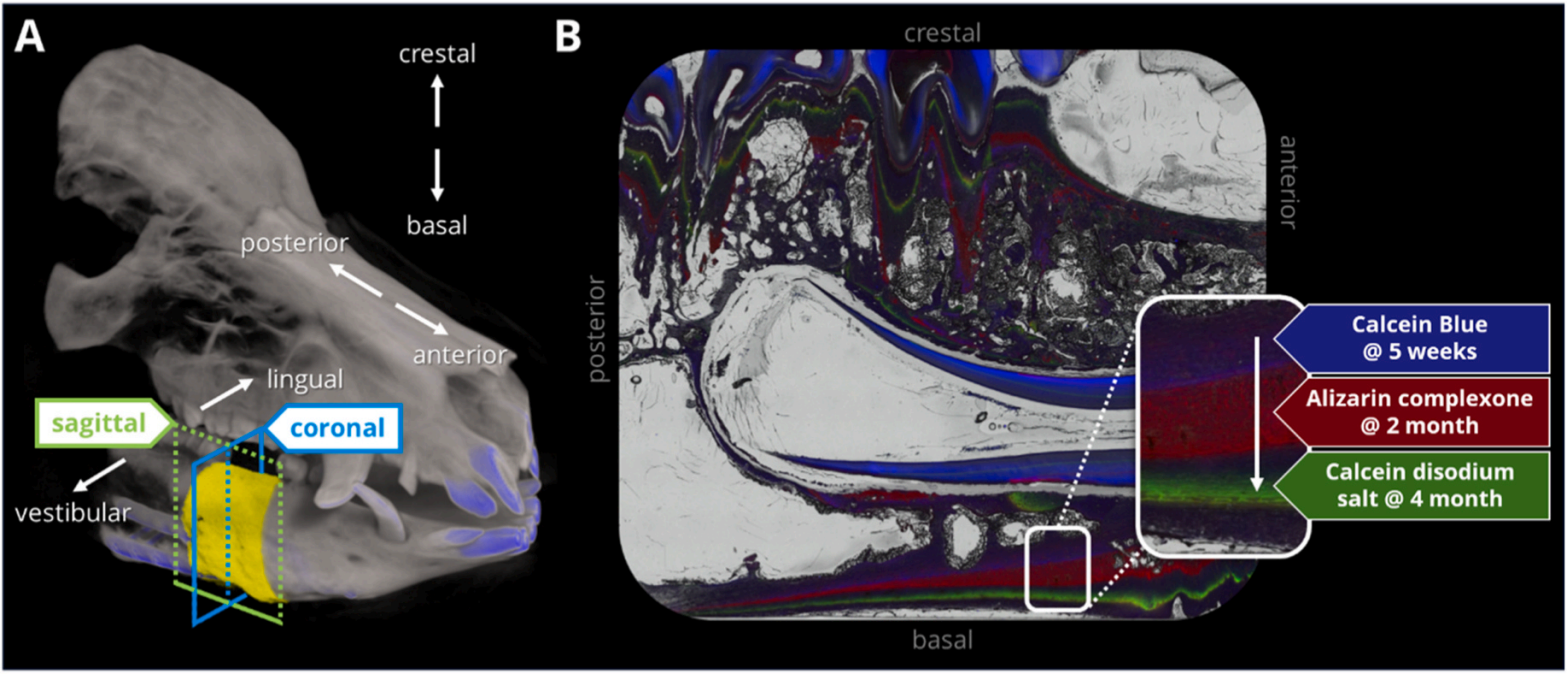
Histological examination with (A) slicing planes (green: sagittal/longitudinal section, blue: coronal/cross-section, yellow: defect area) and (B) thin sagittal section of a healthy untreated left mandible illustrating sequential fluorochrome labelling.

Resected and formalin-fixed mandibles were separated in the middle and then halved again sagitally (Fig. 4A). The vestibular half was used for histological evaluation of the fluorescent dyes (Fig. 4B) in sagittal and coronal overview cross-sections with subsequent histological Masson-Goldner staining. Therefore, specimens were dehydrated in an ascending alcohol series (40 %, 70 %, 80 %, 2 × 96 %, 2 × 100 %), degreased using 2x xylene, embedded in Technovit® 9100 (Kulzer GmbH, Hanau, Germany), grinded to a thickness of approximately 200 μm and polished. After imaging using a Keyence BZ-X800E microscope (Keyence, Osaka, Japan; Calcein Blue: DAPI filter, excitation: 360/40 nm, emission: 460/50 nm; Alizarin complexone: TRITC filter, excitation: 545/25 nm, 605/70 nm; Calcein disodium salt: GFP filter, excitation: 470/40 nm, 525/50 nm), Masson-Goldner staining was performed, which allows better visualization of non-mineralized tissue components [35].

### 2.9 Sample size and statistical analysis

This study was designed as an exploratory pilot study to evaluate the feasibility, safety, and preliminary efficacy of 3D-printed PDLLA-based implants in a large-animal mandibular defect model. Due to the absence of prior data for this specific model and material configuration, no formal *a priori* sample size calculation was performed. Instead, the group size (n = 4 animals per group) was determined based on feasibility considerations, ethical principles to minimize animal use, and consistency with established practice in comparable large-animal pilot studies. All analyzes were predefined prior to data collection and no animals or data points were excluded from analysis.

Statistical analyzes of the numerical results were performed using GraphPad Prism (version 10.5.0, GraphPad Software Inc., USA). Single-time-point comparisons among the three groups used 1-way ANOVA followed by Tukey-adjusted pairwise comparisons (weight increase, Supplemental Fig. A5). Longitudinal DVT outcomes were analyzed using 2-way repeated-measures ANOVA followed by Tukey's multiple-comparisons test comparing implant variants at single time points. Model residuals and Q–Q plots were inspected for gross deviations and influential observations. Formal normality tests were not used to establish normality because of their very low power at n = 4 per group. Results are presented as mean ± standard deviation, and statistical significance was defined as p < 0.05. Given the exploratory nature of the study and limited sample size, statistical analyzes were interpreted descriptively, and emphasis was placed on effect sizes (Cohen's d) and observed trends rather than solely on formal significance testing.

## 3 Results

### 3.1 Postoperative observations

Due to the expected aftereffects of anesthesia and swelling in the surgical area, some of the animals showed drowsy behavior and a reduced food intake for up to 3 days postoperatively. From the 4th postoperative day onwards, a normal general condition was restored. During the 6-months observation period the minipigs showed the expected age-related increase in body weight of 10.9 ± 10.4 % (PDLLA; minimal weight loss in 1 of the 4 animals, animal #1), 7.7 ± 6.8 % (PDLLA + SrHAp-P), and 16.2 ± 3.2 % (PDLLA/SrHAp-C), respectively with no significant differences between the implant groups (Supplemental Fig. A5).

**Fig. 5:**
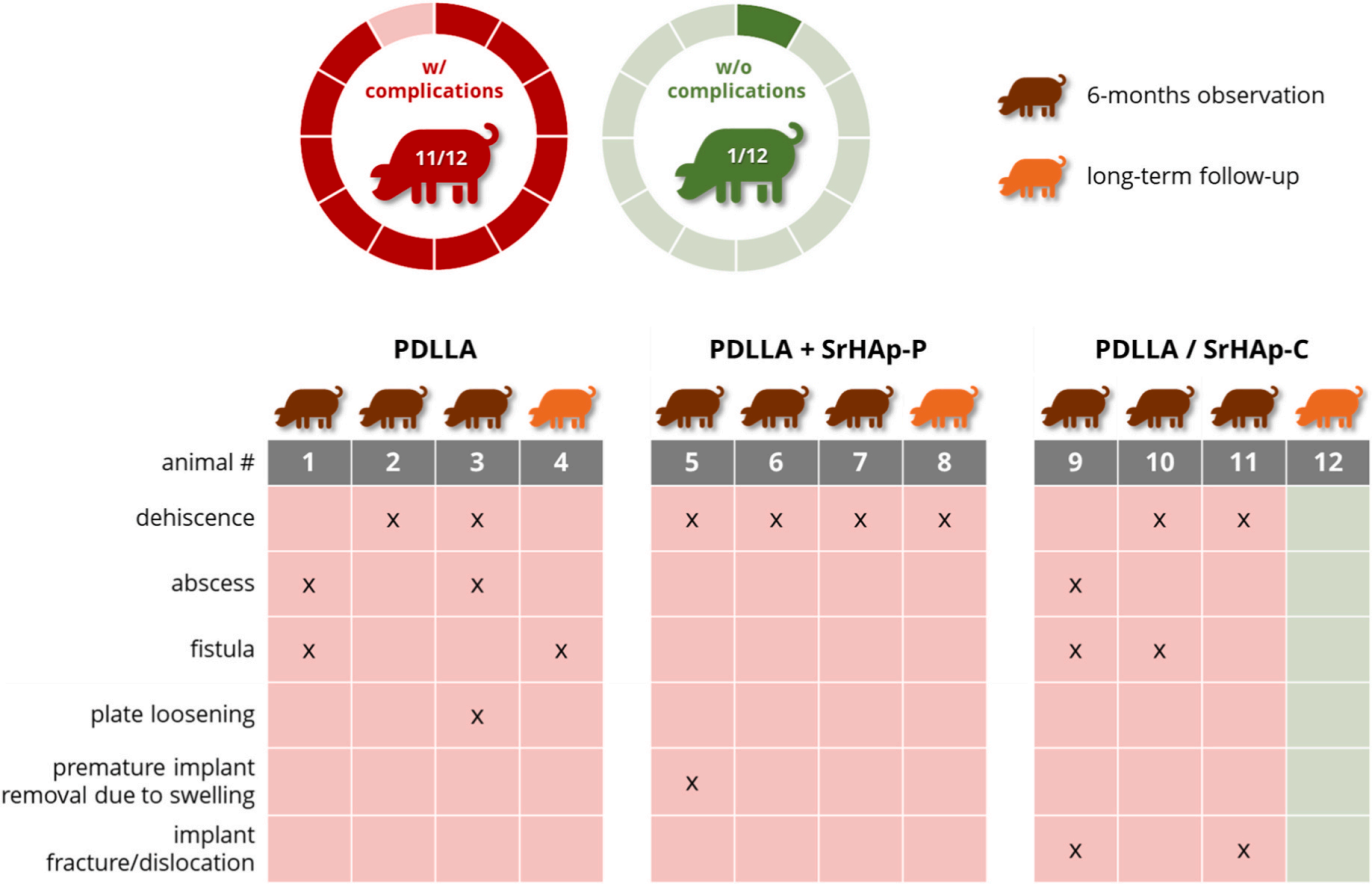
Adverse events per animal during the postoperative observation period. Adverse events occurred in 4/4 animals in the PDLLA group, 4/4 animals in the PDLLA + SrHAp-P group, and 3/4 animals in the PDLLA/SrHAp-C group. Detailed animal-level information, including onset, clinical management, and outcome, is provided in Supplemental Table S1.

Overall, 11 of 12 animals experienced at least one adverse event. A detailed animal-level summary of complication type, onset, treatment, and outcome is provided in Supplemental Table S1. Uncomplicated healing and bone regeneration were observed in only 1 of the 12 minipigs (PDLLA/SrHAp-C, animal #12; Fig. 5). All other animals showed dehiscences, abscesses or fistulas (PDLLA: 4 of 4 animals, PDLLA + SrHAp-P: 4 of 4 animals, PDLLA/SrHAp-C: 3 of 4 animals). In 1 animal of the PDLLA + SrHAp-P group, massive implant swelling necessitated premature removal of the implant after 14 weeks. Within the PDLLA/SrHAp-C group, the SrHAp core fractured and dislocated in 2 animals.

At explantation, complete bone defect bridging was observed in all animals. Additionally, a palpable increase in tissue volume was observed on the lateral and basal sides of the operated area in all animals compared to the opposite side. Intraoral exposure of the reconstruction plate was apparent in 1 of 3 animals of the PDLLA group, in all animals of the composite PDLLA/SrHAp-P, and in 3 of 4 animals of the biphasic PDLLA/SrHAp-C group. While no notable remnants of the PDLLA and composite implant material could be identified macroscopically in any of the 12 minipigs, the SrHAp core was still detectable in 2 of 3 animals of the PDLLA/SrHAp-C group, although it was broken and partially displaced.

### 3.2 Analysis of new bone formation by DVT measurements over 6 months

#### 3.2.1 Qualitative analysis – observations

**Fig. 6:**
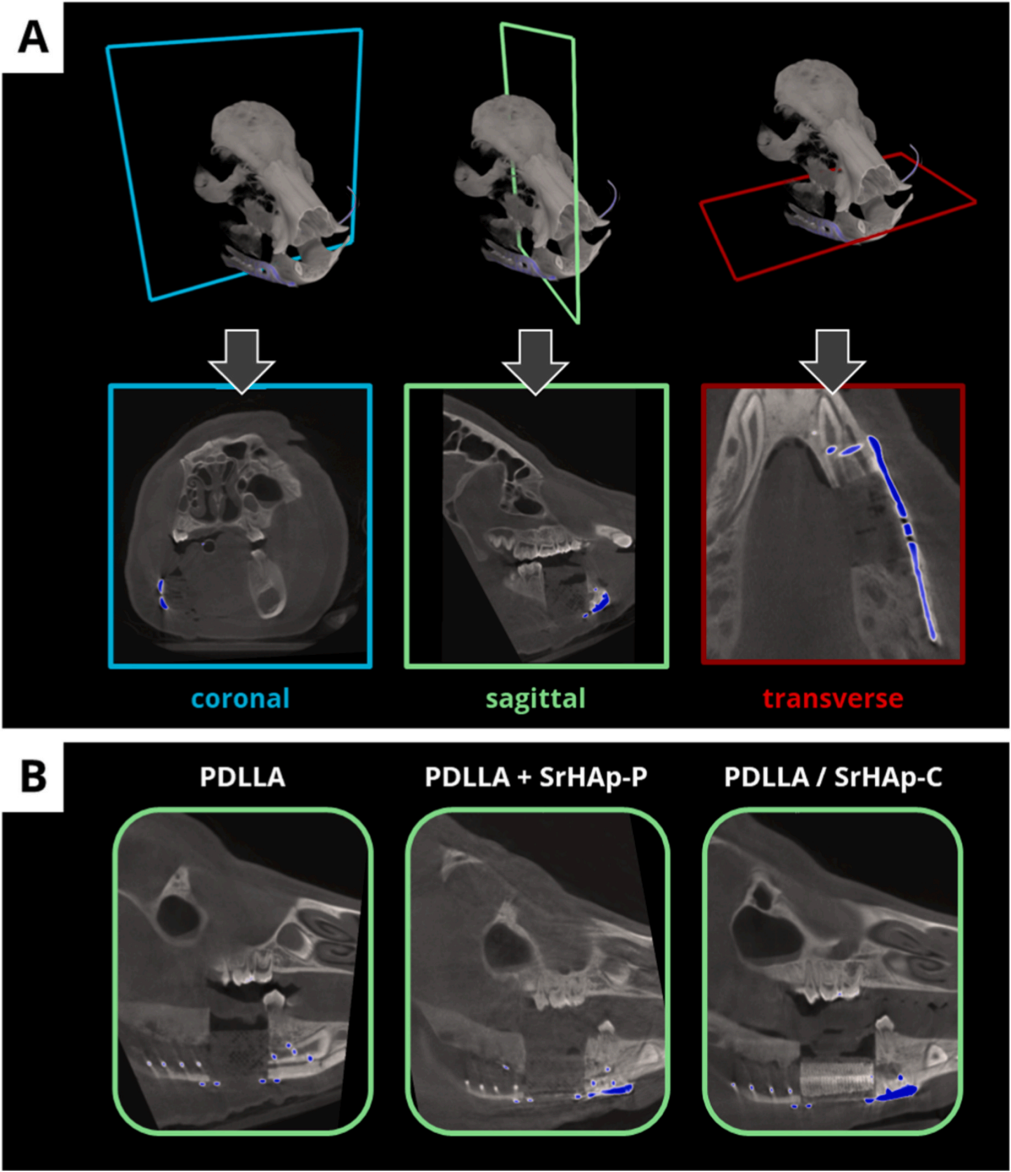
DVT measurement after implantation. (A) Location of the coronal (blue), sagittal (green), and transverse (red) plane in the 3D reconstruction as well as (B) radiographic appearance of the three implant variants immediately after implantation in sagittal view.

DVT images taken immediately after surgery showed clear resection margins, form-fitting reconstruction plates, and a good fit of the implant within the defect with contact to the bone in all animals. The PDLLA and composite PDLLA + SrHAp-P materials were only slightly radiopaque and, in some cases, only visible due to air pockets still present within the implant, whereas the SrHAp core of the biphasic PDLLA/SrHAp-C implant was clearly visible (Fig. 6). The porous lattice structure of the implant was visible for all materials.

**Fig. 7:**
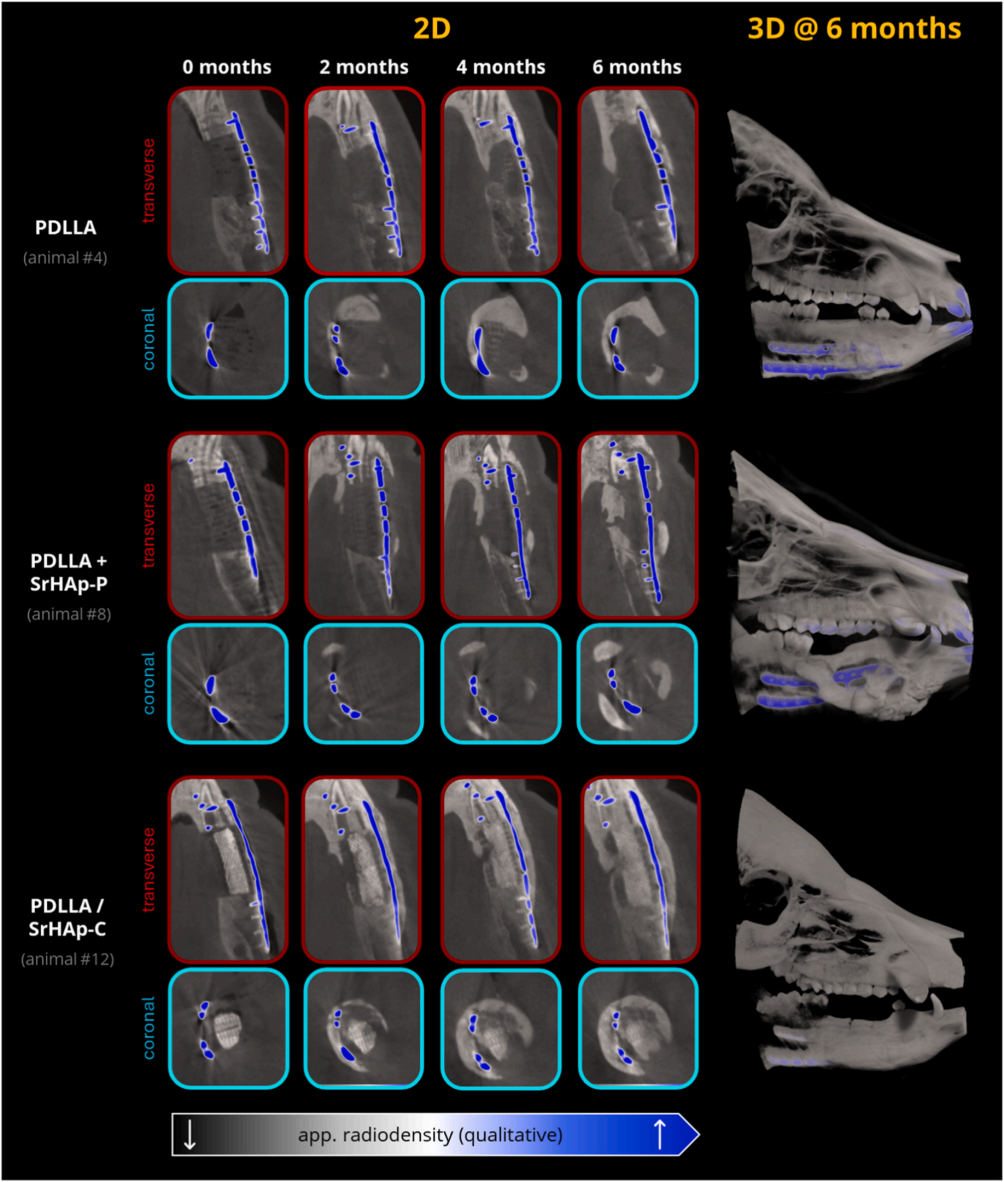
Bone regeneration as evaluated by DVT measurements over time in transverse (red) and coronal (cyan) view as well as DVT-based 3D reconstructions at 6 months postoperatively. Representative results from the animals with the best outcomes that were selected for long-term follow-up are shown; results for all animals are provided in Supplemental Fig. A6–A8. The density color overlay is provided as a qualitative aid for distinguishing structures with different radiopacity and should not be interpreted as a calibrated quantitative density measurement. Images were selected to illustrate changes in implant appearance, position, and surrounding bone formation over time. Images within the same anatomical view were displayed using comparable orientation and field of view (DVT voxel size = 400 μm).

As was already evident during explantation, a bone bridge had formed primarily cranially around the implant across the defect in all animals after 6 months as verified by DVT measurements. New bone formation consistently originated from the periosteum at the peripheral region of the bone stumps and not from the cross-section of the stumps. Sufficient filling of the defect area with newly formed bone was not observed in any animal. Additionally, 3D reconstruction showed that in 10 of 12 animals an additional bone bridge had formed vestibular to the reconstruction plate, either partially or continuously (Supplemental Fig. A6, Supplemental Fig. A7, Supplemental Figure A8, Fig. 7). This bone bridge ran diagonally from posterior-crestal to anterior-basal. Only in one animal of the PDLLA + SrHAp-P and PDLLA/SrHAp-C group, respectively, a homogeneous layer of bone had formed vestibularly over the plate (Fig. 7).

In the PDLLA group, one animal showed anterior osteolysis in the area of the screws and at the posterior bone stump, which led to a slight vestibular dislocation of the plate at 4 months and loss of the plate at 6 months (Supplemental Fig. A6, animal #3). Screw loosening in the anterior region occurred in 3 of 4 animals.

In the composite PDLLA + SrHAp-P group, an almost complete bone bridge had formed cranially to the implant in 3 of 4 animals after 2 months. The remaining animal in this group showed implant swelling and suture dehiscence at 2 months necessitating premature implant removal. Here, a lingual pseudarthrosis persisted until 6 months postoperatively, and the defect remained mechanically unstable (Supplemental Fig. A7, animal #5). Due to extensive osteolysis, the anteriorly adjacent tooth had tilted distally and vestibularly into the defect in 2 of 4 animals. As in the PDLLA group, screw loosening in the anterior region occurred in 3 of 4 animals.

In the biphasic PDLLA/SrHAp-C group, initial bone formation was more heterogeneous in terms of localization than in the other groups. After only 2 months, 1 of 4 animals had formed an almost completely circumferential bone shell around the implant, which had strengthened homogeneously by 6 months postoperatively. The reconstruction plate was osseointegrated and bone ingrowth into the SrHAp core was visible in the lingual area (Fig. 7). Overall, the SrHAp core remained *in situ* until the end of the 6-months observation period in 2 of 4 animals and dislocated in the other 2 animals. In 1 animal, the SrHAp core became displaced after 4 months and was found fractured at 6 months (Supplemental Fig. A8, animal #9) while in the other animal the SrHAp core remained *in situ* until 4 months and dislocated thereafter to the vestibular-submandibular area anterior to the defect (Supplemental Fig. A9, animal #11). Screw loosening was observed in only 1 of 4 animals.

#### 3.2.2 Quantitative analysis of mineralized-tissue volume within the defect-region VOI

**Fig. 8:**
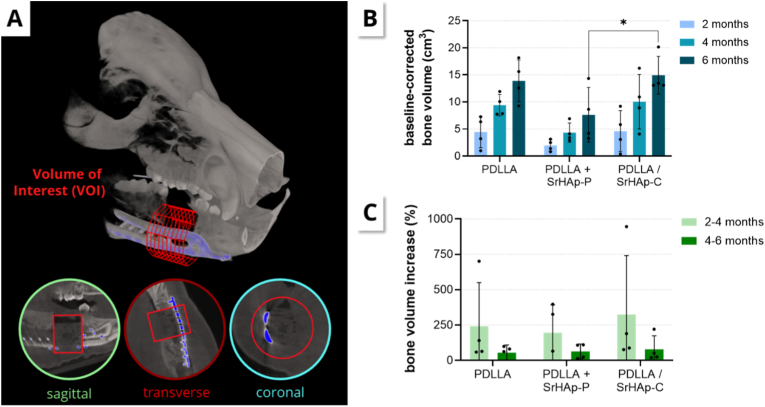
Quantitative evaluation of bone formation by DVT measurements. (A) Volume of interest, (B) baseline-corrected bone volume at 2, 4, and 6 months, and (C) increase in bone volume from 2 to 4 and from 4 to 6 months (mean ± SD, n = 4 animals per group at each time point, *p < 0.05, 2-way repeated-measures ANOVA followed by Tukey's multiple-comparisons test comparing implant variants at single time points).

Over the 6-months observation period, the volume of newly formed bone in the defect area (including periosteal/exostotic bone) increased continuously in all animals, regardless of the implant used (Fig. 8B). At 6 months, animals of the PDLLA/SrHAp-C group showed the highest newly formed bone volume (14.94 ± 3.48 cm^3^) followed by PDLLA (13.90 ± 3.90 cm^3^), and PDLLA + SrHAp-P (7.61 ± 5.06 cm^3^). Based on these results, the effect size (Cohen's d) for PDLLA vs. PDLLA + SrHAp-P is 1.39 and for PDLLA vs. PDLLA/SrHAp-C is 0.28. The highest percentage increase in bone volume occurred in the period between 2 and 4 months (Fig. 8C).

### 3.3 Histologic analysis at 6 months

#### 3.3.1 Dynamic visualization of mineralization by use of fluorochromes

**Fig. 9:**
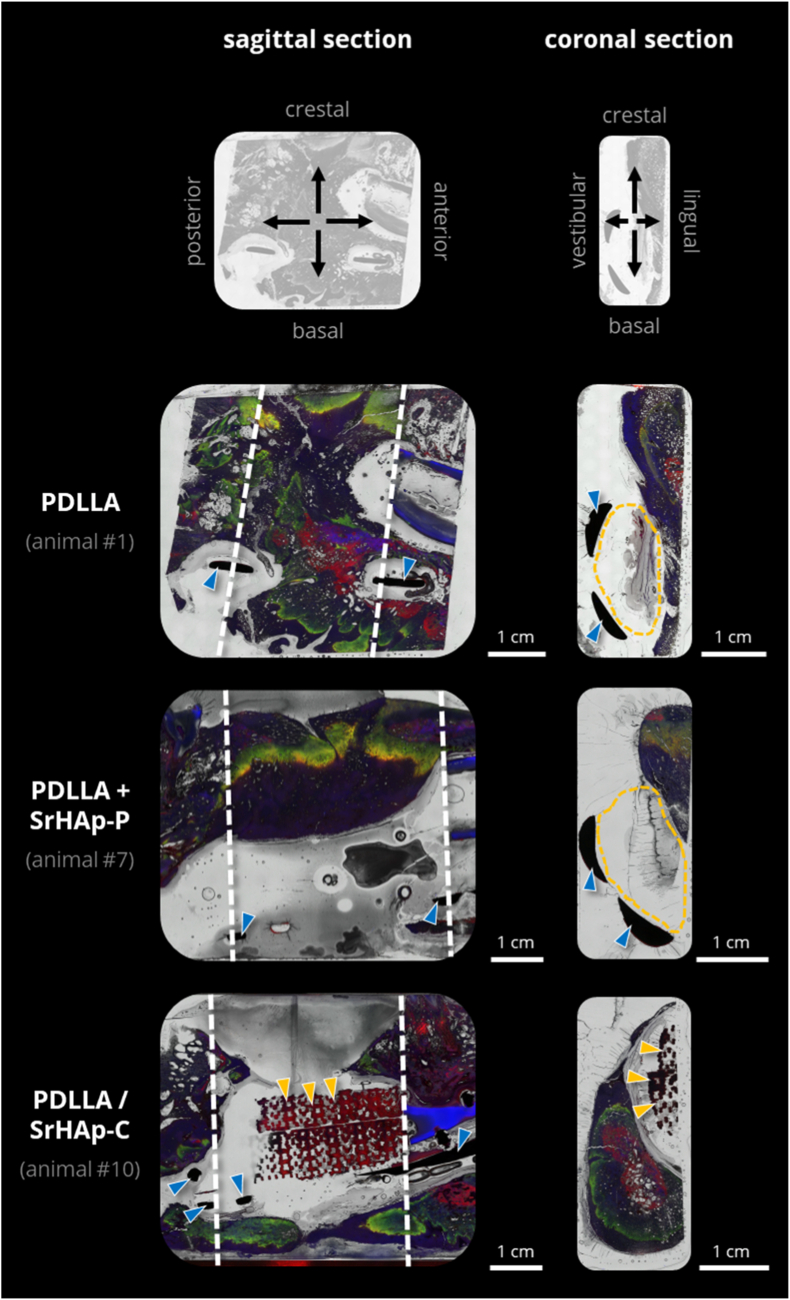
Dynamic visualization of mineralization in the defect area of representative animals by use of fluorochromes that were administered 5 weeks (Calcein Blue – blue), 2 months (Alizarin Complexone – red), and 4 months (Calcein disodium salt – green) postoperatively. White dashed lines indicate the resection margins; orange dashed lines indicate gaps between newly formed bone and the reconstruction plate; blue triangles indicate osteosynthesis material; and yellow triangles indicate remnants of the SrHAp core.

As observed on the healthy contralateral side (see Fig. 4), the chronological sequence of the dyes and thus the new bone formation was clearly seen in the longitudinal section of the defect area, with a blue coloration appearing first from the bone stump or periosteum, followed by a red and green coloration (Fig. 9). The green band of Calcein disodium salt was often followed by an unstained region of bone, indicating formation during approximately months 5–6. In contrast to the healthy side (see Fig. 4), color bands were generally wider due to more extensive bone formation in the defect area as compared to normal bone remodeling at the healthy side (Supplemental Fig. A10). Flat bands of color extending from the bone stumps, indicating bone growth from the resection margins, were rarely seen and comparatively narrow, which indicates slow or inhibited growth (Supplemental Fig. A10). Instead, as could be seen in the sagittal section, the bone bridges often originated with a narrow base at the crestal or basal end of the bone stumps. Bone growth primarily occurred from the crestal to the central area and - in the basal area - primarily to the periphery outside the original periosteal level. This could also be seen in the coronal section: bone growth directed toward the center of the defect originated mainly from the lingual side, while the bone located vestibular to the plate (i.e., new bone formation peripheral to the periosteal level) extended toward the periphery. Additionally, coronal sections of the defect area often showed a clear gap between the formed bone and the reconstruction plate indicating a lack of osseointegration. While remnants of the PDLLA were no longer detectable, the SrHAp core of the PDLLA/SrHAp-C group was still clearly recognizable (Fig. 9).

#### 3.3.2 Masson-Goldner staining

**Fig. 10:**
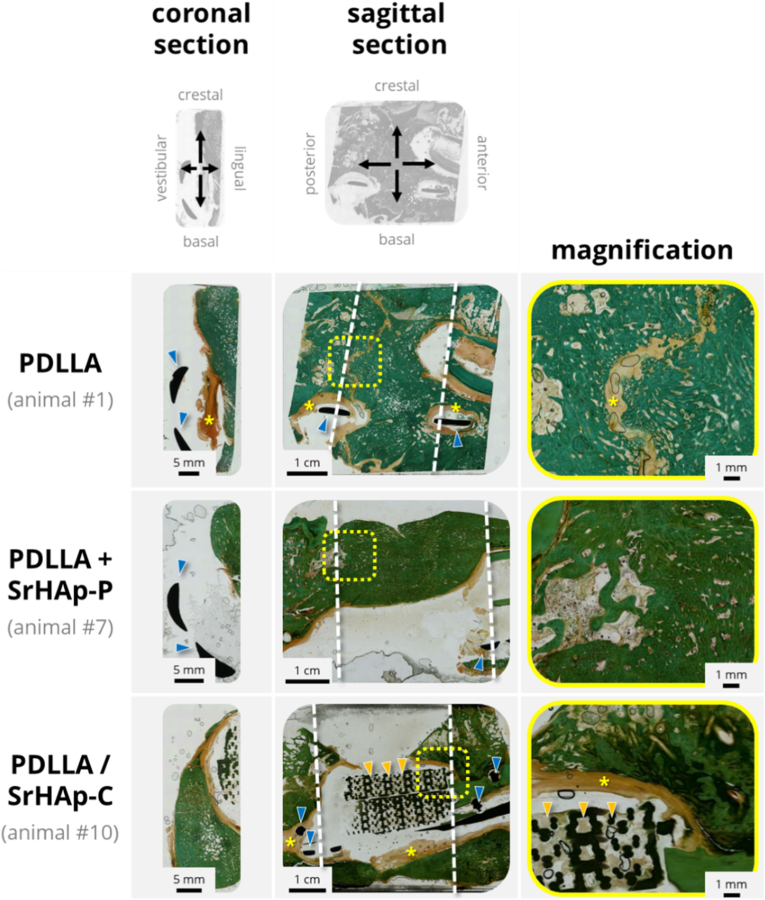
Masson-Goldner staining of the defect area of representative animals. White dashed lines indicate the resection margins; yellow dashed rectangles indicate the area of magnification; blue triangles indicate osteosynthesis material; yellow triangles indicate remnants of the SrHAp core; and yellow asterisks indicate non-mineralized fibrous tissue.

**Fig. 11:**
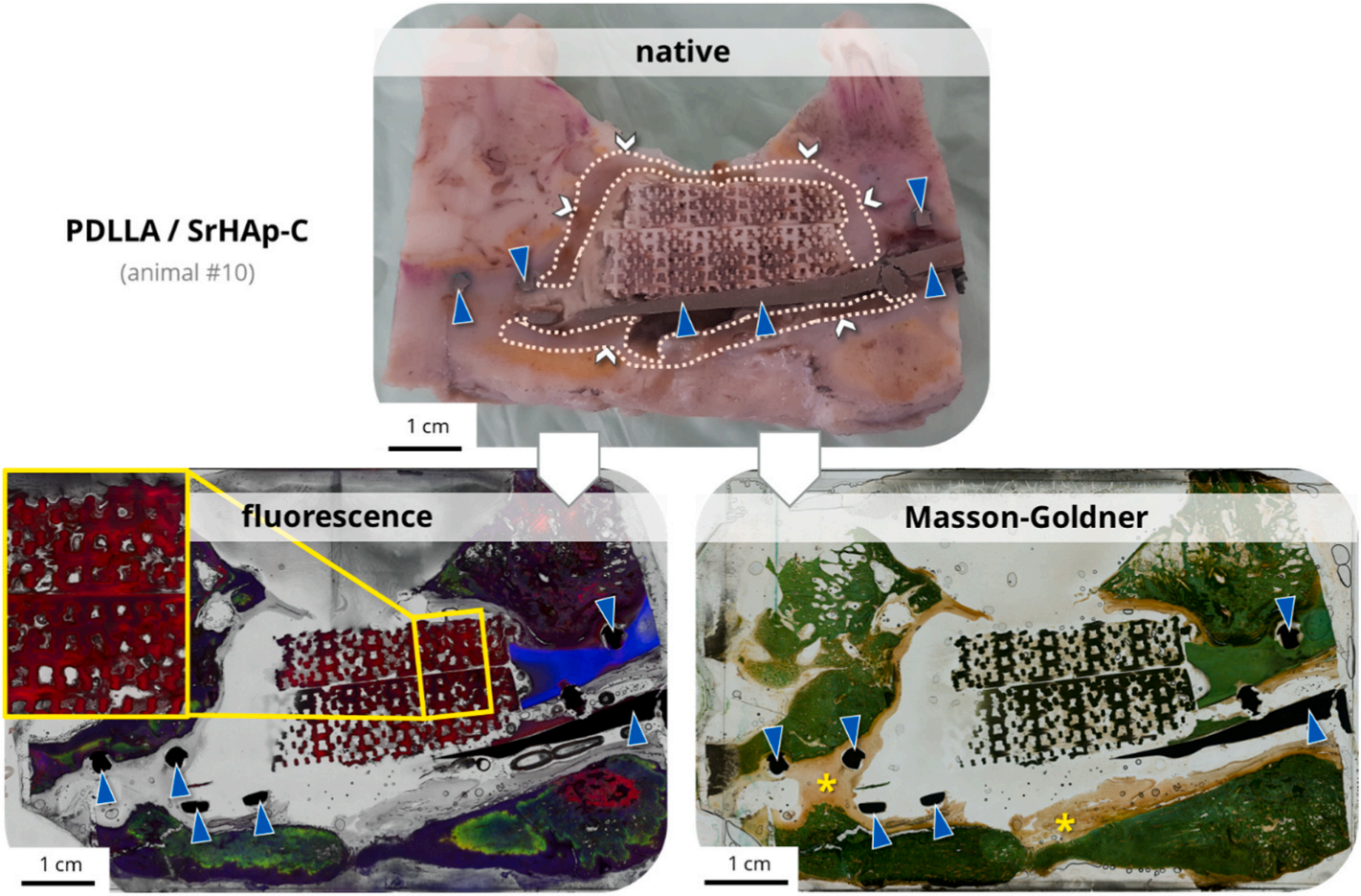
Histologic specimen of an animal of the PDLLA/SrHAp-C group in native condition after explantation and cutting, imaged by fluorescence microscopy and after Masson-Goldner staining. Blue triangles indicate osteosynthesis material; white arrows depicting the dotted area indicate jelly-like material/tissue; and yellow asterisks indicate non-mineralized fibrous tissue.

Non-mineralized fibrous tissue appeared orange-brown and was located at the growth front, e.g., in the gap between two approaching bone ends or around the reconstruction plate (Fig. 10, Supplemental Fig. A11). In contrast to the healthy bone on the untreated left side, the newly formed bone in the defect area was still immature and had a denser structure. The gaps between the bone and the reconstruction plate were clearly recognizable and partially filled with non-mineralized, fibrous tissue. The osteosynthesis screws located further away from the defect were well osseointegrated with close contact between the bone and the screw surface. However, in animals with significantly extended inflammation, some screws adjacent to the defect had lost direct bone contact or were surrounded by fibrous tissue (Supplemental Fig. A11). In the animals of the biphasic PDLLA/SrHAp-C group that were killed after 6 months, the only SrHAp core remaining in the defect was located at the anterior resection margin but showed no tissue ingrowth (Fig. 10). A distinct gap separated the remaining implant surfaces from the surrounding fibrous tissue. This reflects the macroscopic situation during explantation, where a jelly-like material/tissue was present around the core (Fig. 11). The newly formed bone in the defect area showed different stages of bone development, both between the individual minipigs and in some cases within a single defect. The onset of ossification could be recognized by bubble-like cartilage structures and osteoid deposits (chondral ossification, Supplemental Fig. A12). Additionally, areas of immature cancellous bone were visible, recognizable by the still disordered bone matrix and the osteoblasts that were not yet completely enclosed. In terms of surface area, the bone substance still predominated over the medullary cavities. As maturation progressed, osteons with concentrically arranged bone lamellae and embedded osteocytes increasingly formed. Although trabecular formation had increased, the degree of differentiation between cortical and cancellous bone and the degree of trabecular structure of the healthy contralateral side had not yet been reached. In healthy bone, the medullary spaces occupied a greater area relative to the trabecular bone matrix. Cartilaginous areas with incomplete ossification were observed and detailed images of the resection margins showed the transition from the old, organized bone stump to the newly formed, immature, disorganized bone (Supplemental Fig. A12). Clinically visible fistulas were also identified in the histological sections (Supplemental Fig. A13). While the periosteum on the healthy side appeared unremarkable with a narrow osteogenic layer, in the defect area it showed a thickened, more cell-rich, more active osteogenic layer, suggesting increased bone metabolism with enhanced osteogenesis. The fibrous tissue that had grown around the implant remnants (PDLLA/SrHAp-C) and osteosynthesis materials appeared as a homogeneous, cell-poor structure indicating that there was no osteoid as a precursor to osseous integration, but rather a defensive or encapsulation reaction.

### 3.4 Results of long-term observation at 14 months

#### 3.4.1 Prolonged observation time – observations

Eight months after surgery, the minipig that received the PDLLA implant (animal #4) developed an extraoral, weeping fistula that persisted until the osteosynthesis material was removed at month 14. In the animal of the PDLLA + SrHAp-P group (animal #8), the intraoral fistula that appeared 4 months after surgery persisted whereas in the animal of the PDLLA/SrHAp-C group (animal #12), the clinical situation remained unremarkable.

#### 3.4.2 Analysis of new bone formation by DVT measurements

**Fig. 12:**
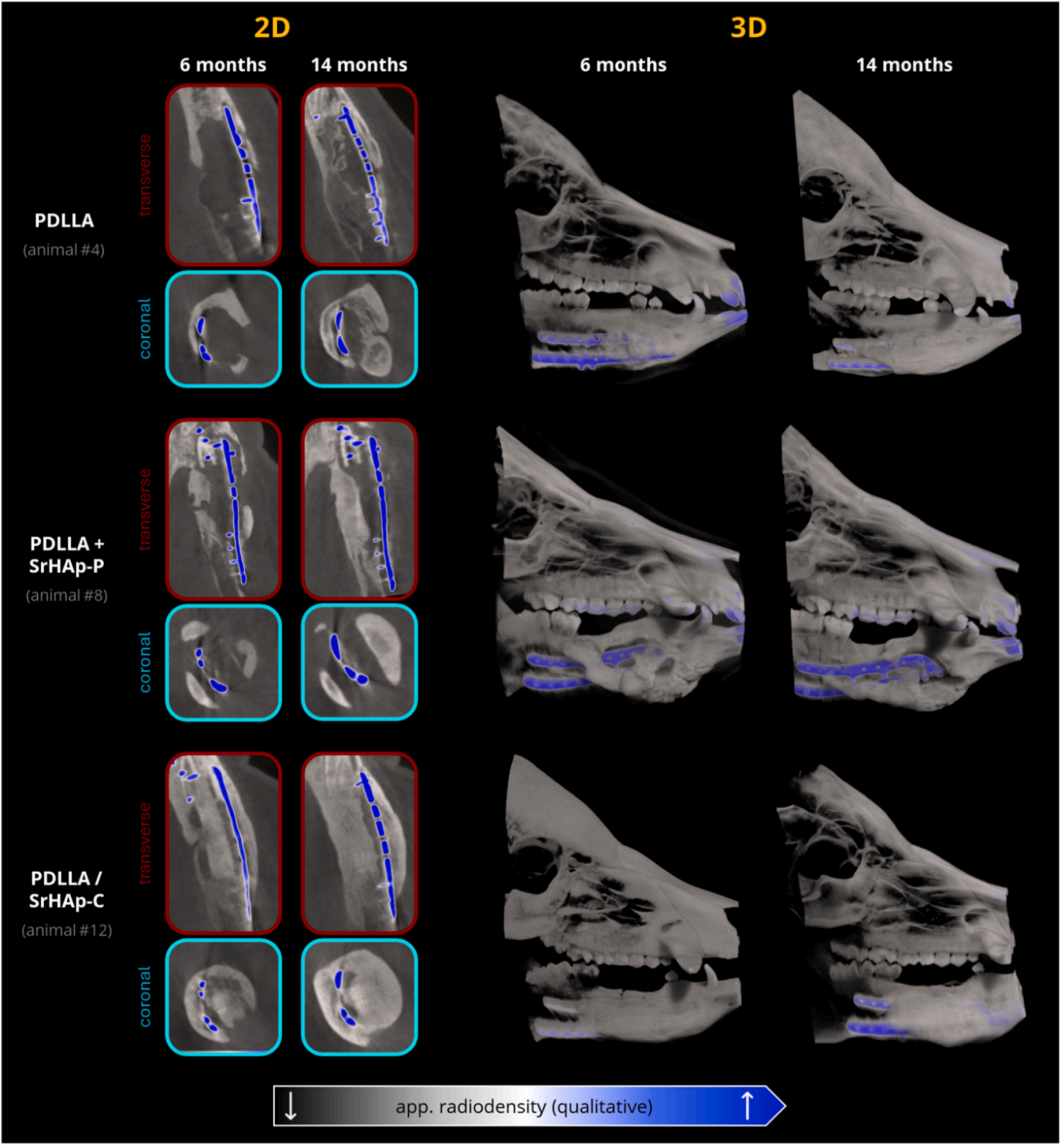
Bone regeneration as evaluated by DVT measurements. Direct comparison of bone growth in the long-term observation period from 6 to 14 months postoperatively in transverse (red) and coronal (cyan) view as well as DVT-based 3D reconstructions at 14 months postoperatively. The density color overlay is provided as a qualitative aid for distinguishing structures with different radiopacity and should not be interpreted as a calibrated quantitative density measurement. Images were selected to illustrate changes in implant appearance, position, and surrounding bone formation over time. Images within the same anatomical view were displayed using comparable orientation and field of view (DVT voxel size = 400 μm).

At month 14, minipig #4 (PDLLA) showed noticeable more bone growth with ingrowth into the former implant area compared to 6 months postoperatively (Fig. 12). Except for a small defect in the lingual area, bone had formed circumferentially around the plate and defect area. The center of the defect was still not completely filled. Compared to the other animals in the PDLLA group, neither exostosis in the submental area nor a vestibular bone diagonal had formed.

In animal #8 (PDLLA + SrHAp-P), one of the 3 separate bone bridges that had formed after 6 months had strengthened after 14 months (Fig. 12). This slightly narrowed the defect area, but did not completely fill it. The vestibular bone diagonal continued to run across the plate at a distance. The loosened anterior screws and the submental exostosis also remained.

In animal #12 (PDLLA/SrHAp-C), the ingrowth of bone into the implant structure, which began after 4 months, had continued (Fig. 12). Particularly in the lingual area, the PDLLA shell was interspersed with bone up to and into the SrHAp core. Only a narrow strip medial to the reconstruction plate was not yet completely ossified. The bone vestibular to the plate was homogeneous and laid directly against it. Neither a submental exostosis nor a vestibular diagonal had formed. The resulting bone bridge was anatomically similar to the original mandible. The SrHAp core showed no clear signs of a beginning resorption.

**Fig. 13:**
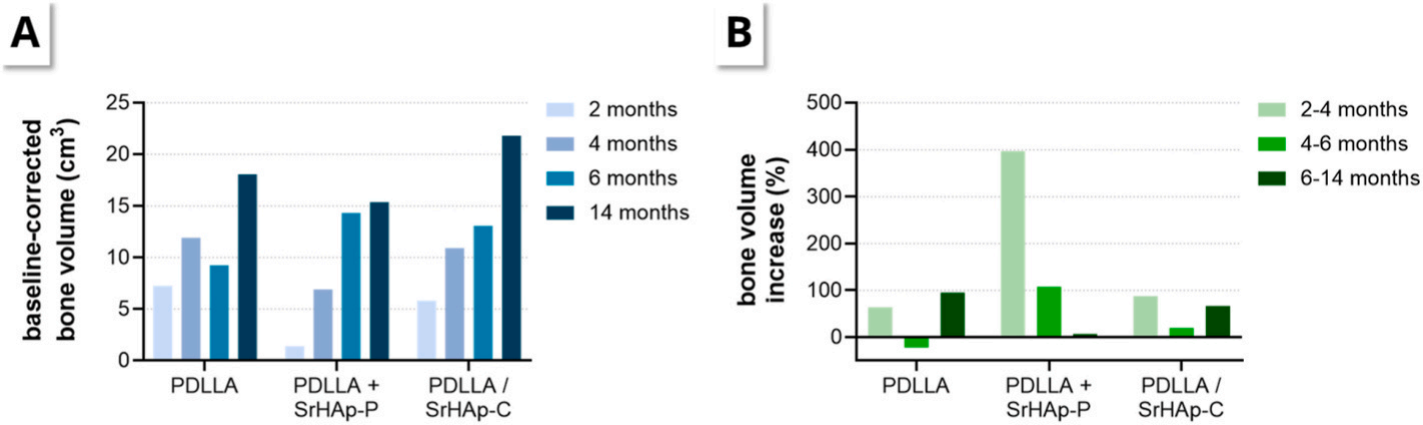
Quantitative evaluation of bone formation by DVT measurements of animals that were observed for 14 months without killing. (A) Baseline-corrected bone volume at 2, 4, 6, and 14 months and (B) increase in bone volume from 2 to 4, from 4 to 6, and from 6 to 14 months (n = 1 animal per group at each time point).

After 14 months of observation, the largest volume of newly formed bone was observed in the PDLLA/SrHAp-C animal (21.82 cm^3^), followed by the PDLLA animal (18.07 cm^3^), and the PDLLA + SrHAp-P animal (15.39 cm^3^) (Fig. 13A). Compared to the 6-months value, bone volume had increased by 95 % in the PDLLA animal, by 67 % in the PDLLA/SrHAp-C animal, and by 7 % in the PDLLA + SrHAp-P animal (Fig. 13B).

**Fig. 14:**
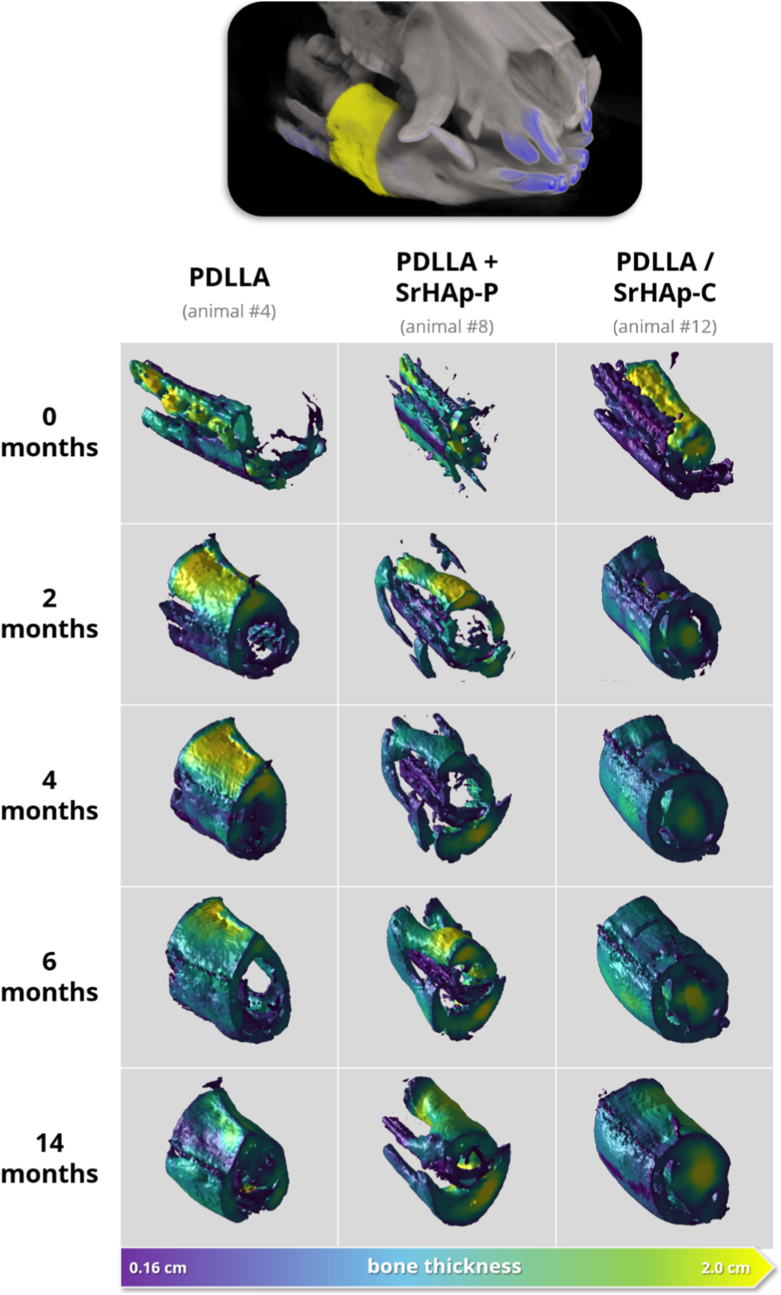
3D reconstruction of the bone (bone thickness) in the defect area based on DVT measurements of the 3 minipigs in the long-term study with a view from the vestibular-anterior-cranial direction (see illustration at top).

DVT-based 3D reconstructions of the new bone formed in the defect area showed the absence of ossification in the center of the defect in the PDLLA animal, as well as interim osteolytic changes after 6 months (Fig. 14, left column). In the PDLLA + SrHAp-P animal, various bone bridges were visible around the implant and the reconstruction plate (Fig. 14, middle column). The PDLLA/SrHAp-C animal showed homogeneous bone formation with osseointegration of the SrHAp core and a physiological three-dimensional anatomy of the mandible (Fig. 14, right column). Here, the only defect remaining after 14 months was a narrow cavity between the SrHAp core and the reconstruction plate.

#### 3.4.3 Removal of osteosynthesis material

As verified by DVT measurements, bone defect bridging was sufficient in all three animals to allow reconstruction plate removal 14 months postoperatively. To remove the screws and the plate, bone that had grown vestibularly over the reconstruction plate had to be removed in all three minipigs.

**Fig. 15:**
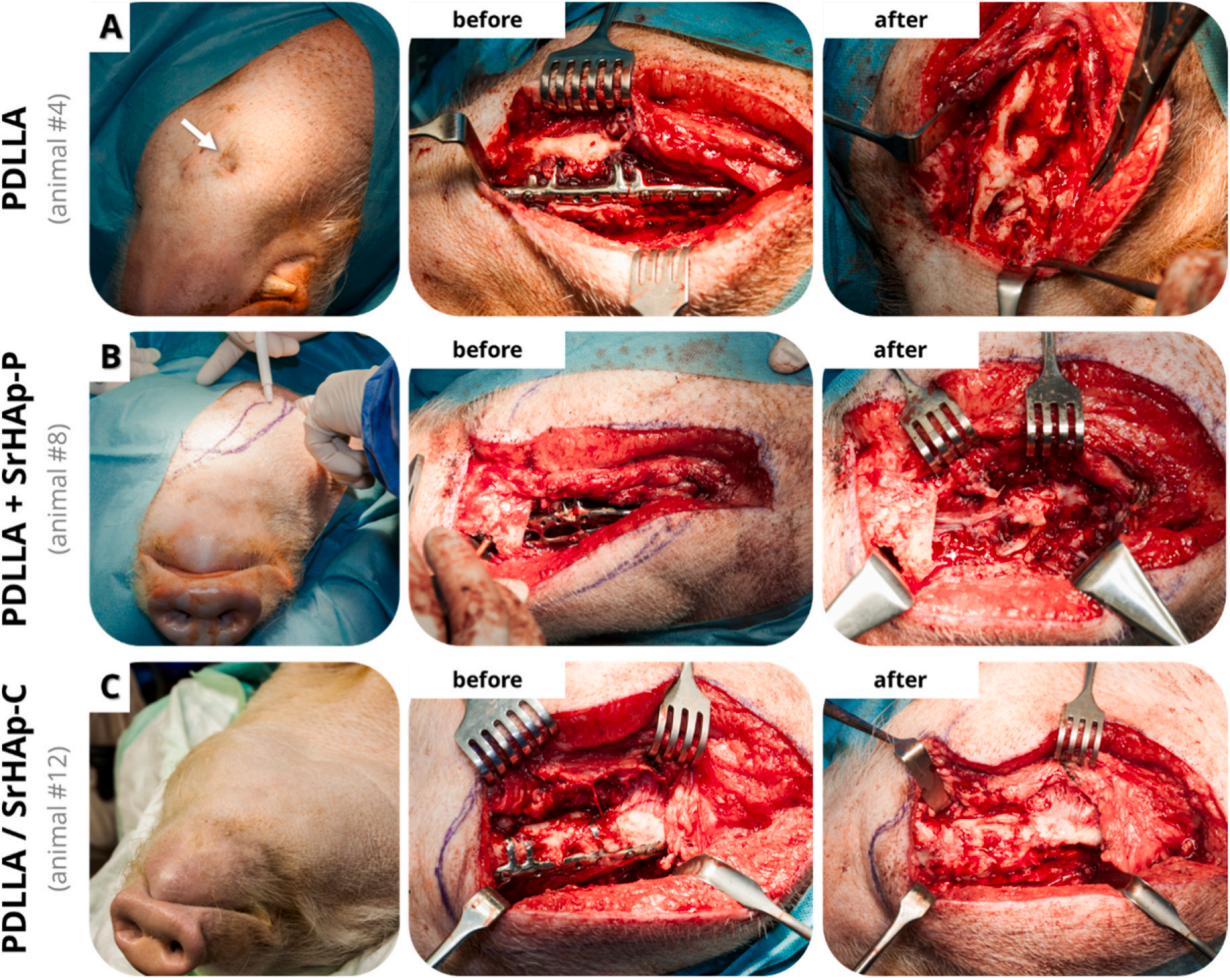
Removal of the osteosynthesis material 14 months postoperatively after DVT-based verification of bone defect bridging. (A) PDLLA (white arrow: persisting fistula), (B) composite PDLLA + SrHAp-P, and (C) biphasic PDLLA/SrHAp-C. Images showing macroscopic view (left column), situation before (middle column) and after (right column) osteosynthesis material removal.

In the PDLLA animal, the fistula persisted extraorally and was therefore resected. The defect was completely bridged from the lingual side except for a gap toward the plate, which was filled with fibrous tissue (Fig. 15A). The underlying bone had an irregular surface with impressions of the retention arms of the plate. Unlike some of the other minipigs, the reconstruction plate did not show any hard deposits. The PDLLA + SrHAp-P animal had an infected defect area connected to the oral cavity, which is why the reconstruction plate had already loosened and showed mineral deposits. Due to the chronic infection, the defect was only slightly bridged compared to the other two animals (Fig. 15B). The PDLLA/SrHAp-C animal showed no extraoral abnormalities and a very well-ossified defect area with no evidence of chronic inflammatory processes (Fig. 15C). After removal of all osteosynthesis material, the surgical sites were closed again in the same way as for the implantation surgery.

## 4 Discussion

In this study, individually manufactured biodegradable implants based on PDLLA were evalu-ated in a minipig mandible defect model. With the aim to buffer its acidic degradation products and to stimulate bone regeneration, PDLLA was combined with strontium-modified hydroxyapatite (SrHAp): The two materials were either combined by mixing set SrHAp microparticles (SrHAp-P) into the PDLLA matrix to form an extrudable composite material (PDLLA + SrHAp-P), or PDLLA and SrCPC paste were extruded separately and subsequently assembled after solidification to build biphasic PDLLA/SrHAp-C implants with a shell/core structure (Fig. 2). During the 6- or 14-months observation period, DVT measurements were performed repeatedly to monitor bone regeneration.

Clinical observation of the animals showed a variety of complications like abscesses, intra-oral suture dehiscences and/or extra-oral fistulas, implant swelling necessitating premature implant removal, or implant fracture/dislocation. Nevertheless, after 6 months, defect bridging could be observed in all animals with formation of new bone starting within the first 5 weeks and originating from the defect borders and the periosteum along the implant/reconstruction plate and around the implant, respectively. Bone ingrowth into the implant could be detected only for the biphasic PDLLA/SrHAp-C. After 14 months, the remaining three animals demonstrated an ongoing bony consolidation with increasing bone volume. Again, new bone formation occurred around the implant, whilst bone ingrowth into the implant could only be seen for PDLLA/SrHAp-C.

The different outcomes of the three implant configurations should be considered in the context of current approaches to the design of additively manufactured polymer-ceramic scaffolds. Recent additive-manufacturing studies demonstrate that scaffold performance can be tuned jointly through architecture and composition. Examples include ion-substituted hydroxyapatite and bioactive-glass-modified triply periodic minimal surface (TPMS) bioceramics, as well as multifunctional PLA- or bio-based polymer scaffolds incorporating ceramic nanoparticles to modulate mechanical properties, degradation, shape-memory behavior, and biological activity [[36], [37], [38], [39], [40]]. Collectively, these studies support an integrated design strategy in which ceramic chemistry, spatial material distribution, porosity, mechanical performance, and polymer degradation are optimized concurrently. However, findings obtained from relatively small scaffolds or short-term defect models cannot be directly extrapolated to large mandibular implants, in which total polymer mass, soft-tissue coverage, fixation stability, and sustained mechanical loading become additional dominant constraints. The behavior of the three implant configurations investigated here is discussed below in relation to these interacting material- and architecture-dependent factors.

### 4.1 Behavior of the implants

#### 4.1.1 PDLLA

The occurrence of dehiscences, fistulas, or abscesses in all animals implanted with PDLLA highlights a significant limitation of this material. In two cases, abscesses were confirmed to be sterile, implicating a foreign body reaction rather than infection as the underlying cause. This observation is consistent with clinical and preclinical reports documenting foreign body reactions – including sterile abscesses and fistulas – in up to 22 % of cases with polylactic acid-based implants, particularly in craniofacial applications [[41], [42], [43]]. Sterile (aseptic) abscesses and fistulas are increasingly recognized as a consequence of the acidic byproducts generated during PDLLA hydrolysis, which can lower local pH and trigger inflammatory tissue reactions even in the absence of infection [42,44]. Inflammation-induced material failure as well as complications such as osteolysis, disintegration, fibrous capsule and cyst formation were also described when using reconstruction plates and screws made of PDLLA [20,45,46].

As shown in a previous study [24], *in vitro* the pH surrounding degrading PDLLA decreased drastically after 12 weeks of incubation, falling below pH 3 after 24 weeks. Compared to the hydrolytic degradation of PDLLA *in vitro*, degradation *in vivo* is promoted by additional enzymatic degradation, phagocytic cells, and mechanical stress with increased erosion and loss of structural integrity and depends also on the implantation site [[47], [48], [49]]. The timing of the complications observed in the present study, with fistulas appearing as early as 5 weeks and abscesses at 6 weeks, 2 months, and 5 months, mirrors the rapid degradation kinetics of PDLLA and the associated local tissue response [50,51]. While rapid resorption is advantageous for avoiding long-term foreign body presence, it can also result in a burst release of acidic byproducts, overwhelming local buffering capacity and precipitating inflammatory reactions [43,44].

Due to the low radiopacity of the PDLLA implants, it proved difficult to differentiate radiologically whether the implants were still intact or degraded. Remnants of the PDLLA could only be detected in one animal where the abscess developed 5 months postoperatively contained crumbly implant fragments. The degradation rate of PDLLA implants varies depending on the implant design and size, site of implantation, and biological conditions. While mechanical stability is often lost within 3–6 months [52], complete resorption typically occurs within 6-12 months [41,52,53]. In the present study, the large volume of the implants (PDLLA: 6.94 ± 1.06 cm^3^, Supplemental Fig. A1) probably led to increased degradation due to autocatalytic properties with accumulation of acidic degradation products, even though the porous structure was designed to prevent this [54]. Possibly, an implantation site with a thicker soft tissue envelope would have better opportunities for pH regulation and the removal of degradation products, as shown, for example, by a study of Heidemann et al. where PDLLA was implanted in the back muscles of rats [41]. Previous *in vivo* studies on PDLLA used smaller samples, different implantation sites, and varied observation periods. For example, small ultrasound-activated PDLLA pins (0.5 × 2.1 mm) implanted into sheep jaws showed no adverse findings after 9 weeks [55]. Larger PDLLA cylinders (Ø 6 mm, length: 15 mm) implanted in dog back muscles remained free of infection or inflammation for up to 12 months [56]. Similarly, pre-degraded PDLLA implanted intramuscularly in rats caused swelling only in the first 4 weeks, with observation lasting up to one year [57]. Resorbable PDLLA plates (thickness: 1 mm) and screws (Ø 2.1 × 4 mm) were used in craniosynostosis surgeries, with swelling occurring after 3, 9, and 12 months [58]. Similar PDLLA materials (plates: 2 mm thick; screws: Ø 2 mm) did not cause any significant complications in sheep after condylar neck fractures in the mandible after 12 months [59]. In human patients, inflammatory reactions occurred in 4 % of cases after 4–20 months when PDLLA plates and screws with the same dimensions were used [45].

Despite defect bridging was observed after 6 months, complete filling of the defect area with new bone was not achieved in any of the 4 animals. Histological and radiological analyzes revealed that new bone formation originated primarily from the periosteum, with minimal contribution from the bone stumps and no evidence of bone ingrowth into the PDLLA implant. This pattern is well-documented in the literature since the periosteum is a critical reservoir of osteoprogenitor cells and growth factors [[60], [61], [62]]. Thus, bone formation typically proceeds along the reconstruction plate and periosteal surfaces, but rarely results in complete defect closure, especially with biodegradable polymer scaffolds [63,64]. Moreover, as shown by DVT measurements at 14 months postoperatively, PDLLA can sustainably compromise physiological bone regeneration/remodeling that was also found in other studies [65,66]. The absence of bone ingrowth into the PDLLA implant further highlights the limitations of this material in supporting osteoconduction in large defects. Previous studies have shown that even with optimized scaffold architecture, bone ingrowth into PDLLA or PLGA-based scaffolds is limited unless combined with osteoinductive coatings or cell seeding [63,67]. As evident from the formation of the vestibular bone bridge in 3 of the 4 animals, the mandible as an implantation site also posed a significant mechanical load despite stabilization by the reconstruction plate. This could have further weakened the structure of the implants.

#### 4.1.2 PDLLA + SrHAp-P composites

As for PDLLA, wound dehiscences, abscesses, or fistulas were observed in all animals of the PDLLA + SrHAp-P group, including one case necessitating premature implant removal 14 weeks postoperatively due to massive swelling. These observations indicate a severe foreign body and/or degradation-associated inflammatory response and are consistent with previous reports that the addition of calcium phosphate particles to PDLLA, such as tricalcium phosphate (TCP) or hydroxyapatite (HAp), can exacerbate local inflammatory reactions and sterile abscess formation, likely due to the combined effects of acidic degradation byproducts and persistent particulate debris [41]. Although previous *in vitro* investigations stated a good buffering capacity of PDLLA blended with SrHAp particles, with a pH of approximately 6 after 24 weeks [24], the current results suggest that *in vivo* the addition of SrHAp-P in the relatively small amount used here (mass ratio PDLLA:SrHAp = 90:10) failed to sufficiently neutralize degradation effects and prevent adverse tissue reactions. Additionally, water uptake and autocatalytic degradation might have promoted rapid mass swelling and soft tissue irritation. Composites made of HAp and poly(L-lactide) (PLLA) have been shown to exhibit accelerated degradation compared to the pure polymer, due to increased water diffusion pathways [68,69]. This is in line with results of our previous *in vitro* study where PDLLA + SrHAp-P showed increased swelling over 24 weeks compared to pure PDLLA [24]. That was considered acceptable, but unlike *in vitro*, 3-dimensional swelling was limited in 5 out of 6 directions *in vivo*: anteriorly and posteriorly by the bone stumps, vestibularly by the reconstruction plate, caudally by the surrounding muscle tissue, and lingually by tongue pressure. Thus, swelling was concentrated primarily cranially, where the intraoral sutures were the weak points.

The absence of macroscopically detectable implant remnants at explantation (beside an implant fragment that was trapped between the reconstruction plate and the mucous membrane in one animal) strongly indicates rapid *in vivo* degradation, which appears to have outpaced the regenerative process. Experimental studies on PLLA/HA composites demonstrate that HA incorporation can accelerate polymer degradation and molecular weight loss [69]. Xiao et al. implanted PDLLA/HA rods into the femora of rabbits and observed substantial degradation of the composite occurring at 8 weeks that led to the formation of pores and structural weakening [70]. While such pore formation is often considered beneficial for tissue ingrowth, this is only advantageous if degradation is gradual, and mechanical integrity is maintained during early healing phases. In the present study, the data suggest a loss of space-maintaining function at an early stage, consistent with implant swelling, intraoral plate exposure and screw loosening.

Despite adverse conditions, early cranial bone bridging (3 of 4 animals at 2 months; for comparison: 2 of 4 animals at 2 months for PDLLA) was observed. However, regeneration was characterized again by periosteum-driven bone formation along the reconstruction plate and implant surface and limited contribution from the bone stumps. This pattern indicates that bone regeneration occurred largely independently of the implant, relying on external biological sources rather than scaffold-mediated osteoconduction. This is notable because HAp-containing composites are generally considered osteoconductive. However, this effect may depend on the amount and accessibility of HAp present in the composites. While in other studies demonstrating greater osteoconductivity the HAp content of the composites was approximately 40 %, PDLLA composites in our study contained only 10 % of SrHAp [71]. Furthermore, literature clearly shows that osteoconductivity depends critically on scaffold architecture, particularly surface accessibility of HAp and interconnected porosity. Since the macroporous structure of the implant was altered by swelling, which resulted in pore obstruction (Supplemental Fig. A14), osteoconduction and bone ingrowth was further limited. Thus, the implant likely remained functionally dense during early healing, preventing cell migration. Comparison of the newly formed bone volume between PDLLA and the composite PDLLA + SrHAp-P revealed a large effect size (Cohen's d ≈ 1.39), indicating a substantial reduction in bone formation in the particle-based group. However, given the limited sample size and variability, this finding should be interpreted as an exploratory trend. Although partial defect bridging was observed, complete defect filling was not achieved, even after 6 months. This outcome reflects a classic failure mode in biodegradable implants, where degradation outpaces tissue formation and the implant fails to provide long-term guidance and stability. Comparative studies confirm that even well-designed PDLLA/HAp implants may show inferior bone regeneration compared to alternative systems (e.g., PLGA/HAp), particularly when degradation kinetics are not optimal [72]. As also seen for PDLLA, all animals of the PDLLA + SrHAp-P group developed a vestibular diagonal bone bridge, indicating a suboptimal load transfer from the reconstruction plate to the mandible.

In the present study, PDLLA + SrHAp-P implants did not support functional defect regeneration demonstrating that the primary limitation is the intrinsic coupling of degradation kinetics (insufficient buffering, swelling and inflammatory response) and implant architecture (lack of macroporosity and interconnectivity due to clogged pores, absence of osteoconductive guidance).

#### 4.1.3 Biphasic shell/core PDLLA/SrHAp-C

In the biphasic PDLLA/SrHAp-C group, soft tissue complications were observed in 3 out of 4 animals, including dehiscences, abscesses, fistulas, and intraoral exposure of the reconstruction plate. As already discussed for PDLLA and PDLLA + SrHAp-P, this indicates a severe foreign body and/or degradation-associated inflammatory response.

Radiological investigations at 6 months showed that the SrHAp core was still detectable in all 4 animals (Supplemental Fig. A8), even though the core remained within the original defect area in only two animals. In one of these two animals, the SrHAp core showed signs of osseointegration (animal #12) while in the other animal (animal #10) the core was surrounded by a jelly-like material identified by Masson-Goldner staining as fibrous tissue that is characteristic for a foreign-body response. Dislocation of the implant was observed in the two remaining animals, once towards lingual (animal #9, implant fractured without function) and once towards submandibular (animal #11, SrHAp core osseointegrated outside the defect area, Supplemental Fig. A9). This highlights a critical mechanical weakness at the PDLLA shell – SrHAp core interface. While PDLLA degrades fast leading to a reduced shell stability, the SrHAp is quite brittle with insufficient tensile strength. This leads to stress concentration, insufficient load transfer with possible fracturing, particularly under mandibular loading conditions.

In contrast, degradation of the SrHAp is quite slow since remnants of the SrHAp core were still visible radiologically after 14 months. In a study conducted by Kuang et al. cylinders made of CPC and CPC containing 10 % strontium were tested in a rat femur bone defect model [73]. Although the strontium addition enhanced osteoconductivity and accelerated the degradation rate, complete resorption was not achieved even 32 weeks after implantation. The degradation of 3D-printed calcium phosphate-based cements is determined also by the biological environment/implantation site and mechanical stress [74]. In a retrospective study conducted by Richter et al., macroporous 3D scaffolds printed from a calcium phosphate cement paste (CPC) were clinically used for open wedge high tibial osteotomy in 55 patients [75]. After 12 months, complete osseointegration was observed in all patients and although still visible, radiographically the volume of the wedge had significantly decreased indicating substantial resorption. Factors potentially contributing to the enhanced resorption observed in these clinical cases include the specific defect type (open wedge vs. segmental in our study) and, as a result, better anchoring within the defect; the absence of adjunctive materials such as PDLLA, which may impede bone regeneration and weaken structural stability; and the increased mechanical load at the implantation site leading to a more optimal force transmission to the implant. If degradation is too slow (like in our study for the SrHAp core) natural bone remodeling is negatively affected by hindering or even blocking vascularization as well as inhibiting a gradual transfer of load from the implant to the regenerating bone.

Despite complications, all animals achieved complete bone defect bridging, with the highest newly formed bone volume in the defect region among all groups. Compared to PDLLA this effect was very small (Cohen's d ≈ 0.28), but since effect size reflects only total volume, qualitative advantages in osteointegration due to the biphasic scaffold design are not fully captured by bulk volumetric measures. The observation of cranial bridging across the defect supports the notion that bone regeneration was guided more by biological gradients and vascular supply than by the internal architecture of the implant. A vestibular diagonal bone bridge as observed also for the two other implant types, was present in 3 of 4 animals suggesting an insufficient load transmission by the reconstruction plate. Bone ingrowth into the SrHAp core was observed in the lingual area, and one animal developed an almost circumferential bone shell around the implant by 2 months, which matured by 6 months. This temporal pattern is consistent with the known behavior of calcium phosphate-based biomaterials, which provide rapid osteoconduction during the early phases of healing [76]. The presence of strontium within the hydroxyapatite phase may have further enhanced this effect, as strontium ions are known to stimulate osteoblastic activity while simultaneously inhibiting osteoclastic resorption [29,32,33,77,78]. These dual effects could explain the substantial bone formation observed despite the mechanical and biological complications associated with the implant. As for PDLLA and the composite PDLLA + SrHAp-P, bone formation was primarily periosteum-driven with limited contribution from the bone stumps highlighting the role of the periosteum in mandibular defect models. Bone ingrowth into the SrHAp core (animal #12) and partial osseointegration (animal #11) confirms osteoconductivity of the SrHAp as well as preservation of biological functionality despite fragmentation. Another factor influencing bone ingrowth and thus the degradation/resorption of the implant, is its structure/geometry. Korn et al. found a significant influence of the pore geometry (60° > 30° rotated layer orientation) for 3D-printed CPC scaffolds implanted in a rat alveolar cleft model with respect to osteoconductivity [79]. Pore size was investigated by Wu et al. [80] who evaluated the bone regeneration performance of 3D-printed bioceramic scaffolds with varying pore sizes (200 to 600 μm) in a critical-size femoral defect model in rabbits. Results showed that larger pore sizes (450 and 600 μm) significantly enhanced early and extensive bone tissue ingrowth and remodeling compared to smaller pores. Since the pores of the implants used in our study were in the range of 600-1300 μm, this is unlikely to be the reason for the insufficient bone ingrowth observed. It is more likely that the foreign body reaction or inflammation triggered by the rapid degradation of the PDLLA shell prevented cellular infiltration and vascularization in the early stages, as bone ingrowth was not detectable until 4 months after implantation.

Several non-exclusive mechanisms may also explain the local bone ingrowth observed in the biphasic PDLLA/SrHAp-C group. First, replacing part of the PDLLA volume with a contiguous SrHAp core reduced the total amount of acid-generating polymer and may therefore have attenuated the degradation-associated inflammatory response (Supplemental Fig. A1, biphasic implant contained approximately only 50 % PDLLA as compared to the other 2 implant types). Second, the spatially separated and directly exposed ceramic phase provided a more accessible osteoconductive surface than SrHAp microparticles embedded within the PDLLA matrix, which may remain partly inaccessible until polymer degradation occurs. Third, the biphasic architecture may have better preserved the connectivity of the ceramic pore network and enabled more localized buffering of acidic degradation products. However, these explanations remain hypothetical because local pH, strontium-release kinetics, temporal changes in porosity, and early inflammatory responses were not directly measured. Moreover, fracture and displacement of the SrHAp core in 2 animals demonstrate that increasing the ceramic fraction alone is insufficient without improved mechanical integration between the polymer shell and ceramic core.

Thus, the biphasic configuration showed potentially favorable local osteoconductive properties, but these benefits were offset by mechanical instability, soft-tissue complications, and heterogeneous biological outcomes.

#### 4.1.4 Mechanical implications of the implant architectures

The mechanical properties of the three implant architectures were not experimentally determined in the present study, which limits interpretation of their architecture-dependent biological responses. The pure PDLLA implant was expected to provide a continuous polymer network, but hydrolytic degradation and swelling may have progressively reduced its stiffness and dimensional stability. Incorporation of SrHAp microparticles may have altered the stiffness and brittleness of the PDLLA matrix; however, swelling and pore obstruction likely compromised its space-maintaining function and altered local load transfer. The biphasic shell/core configuration introduced a mechanically discontinuous interface between the polymer shell and the comparatively brittle ceramic core. Fracture and displacement of the SrHAp core in two animals indicate insufficient mechanical integration of the two phases. Although the titanium reconstruction plate was intended to provide the principal load-bearing function, differences in scaffold stiffness, structural integrity, and coupling to the plate may have affected micromotion, preservation of the pore architecture, soft-tissue irritation, fibrous encapsulation, and bone ingrowth. Future studies should therefore include compression, bending, interfacial-strength, and cyclic-fatigue testing under physiological wet conditions and at defined stages of degradation.

In addition to the intrinsic mechanical behavior of the implants, the fixation concept and the transfer of load between the scaffold, reconstruction plate, and residual mandible are likely to have influenced the observed regeneration pattern.

#### 4.1.5 Aspects pertaining to all implants

As already mentioned, 10 of 12 minipigs developed an additional vestibular diagonal bone bridge partially or completely. Its shape indicates that the mandible was still exposed to considerable mechanical stress despite stabilization using a reconstruction plate. Thus, the design of the reconstruction plate used in the present study seemed to be suboptimal, as evidenced by the frequent occurrence of screw loosening, particularly at the anterior bone stump. Ma et al. observed similar screw loosening when using two horizontally arranged reconstruction plates that were not connected to each other [81]. This promoted instability of the osteosynthesis and thus additional bone bridge formation.

Histological analysis revealed a distinct accumulation of the applied fluorescent dyes corresponding to both the chronological sequence and anatomical localization of bone formation. The observed pattern of bone growth indicated that the majority of new bone originated from the periosteum adjacent to the defect site. This finding highlights the pronounced osteogenic capacity of the periosteal tissue in the context of defect healing. Conversely, minimal bone regeneration was observed emanating from the bone stumps. Similar observations were reported by Lemperle et al. [82], who demonstrated that, following continuity resection of the mandible in dogs, bone regeneration predominantly occurred on the periosteum-covered side, with only limited contribution from the bone stumps. Additional bone formed along the reconstruction plate and around the exterior of the implant, indicating a lack of osteoconductivity of the implant. Moreover, the PDLLA appeared to impede new bone formation, as evidenced by the absence of bone regeneration at the initial implant site, even after premature removal of one implant (PDLLA + SrHAp-P, animal #5). This suggests that the presence of the implant may have negatively influenced the local osteogenic environment, thereby inhibiting bone healing in that region. This is in line with findings of Kauffmann et al. [83] where bone defects were created in the alveolar crest of the maxillae of minipigs and left empty (SHAM) or filled with PDLLA/CaCO_3_ particles. After 4 weeks no signs of induced bone formation between the PDLLA/CaCO_3_ particles and they were separated from the underlying native bone by a fibrous tissue layer. Compared with sham-treated animals, animals treated with PDLLA/CaCO_3_ showed less newly formed bone indicating an inhibitory effect on bone regeneration.

In the present study, bone formation from the stumps was further impeded by the presence of acute and chronic inflammation. In the initial stage of defect healing after osteotomy, there is always an inflammatory phase characterized by the release of proinflammatory mediators and growth factors. These signals lead to the recruitment of inflammatory cells and mesenchymal stem cells and to the initiation of angiogenesis. In this context, the periosteum is activated, which supports bone regeneration [84,85]. The inflammatory response is therefore an essential step in the physiological process of bone healing. In contrast, an excessive inflammatory response, e.g., due to infection or a foreign-body reaction, causes excessive production of signaling molecules, which activate osteoclasts. If the triggers of acute inflammation cannot be eliminated, chronic inflammation develops, which can also lead to inhibition of osteoblasts and activation of osteoclasts, thereby promoting bone resorption [84]. In the present study, these processes were observed in the minipigs when acute abscesses led to osteolysis of already formed bone segments (e.g., PDLLA: animal #3 and #4) and chronic inflammation caused by suture dehiscence led to significantly reduced bone formation (e.g., PDLLA + SrHAp-P: animal #6 and #7).

Prolongation of the observation period up to 14 months revealed an ongoing bone formation and remodeling leading to a bone volume increase in all 3 animals (PDLLA: 95 %, PDLLA/SrHAp-C: 67 %, PDLLA + SrHAp-P: 7 %).

### 4.2 Implant design and defect model

The regeneration of bone defects in the oral and maxillofacial region poses a particular challenge due to the complex anatomy and high functional requirements. With its anatomical and physiological similarity to the human mandible - including comparable bone structure, density, and remodeling patterns, the minipig mandible defect model is particularly suitable for preclinical studies in craniomaxillofacial research [86]. The model provides a large and clinically relevant segmental mandibular defect for assessing implant performance under complex mechanical, inflammatory, and soft-tissue conditions. Nevertheless, preservation of the periosteum maintained substantial endogenous regenerative capacity, as reflected by defect bridging even in the animal that underwent premature implant removal; therefore, the model cannot be classified as a strict critical-size defect. Furthermore, the oral environment and masticatory forces in minipigs closely mimic those in humans, providing a realistic setting for testing implant stability and bone healing under functional loading [61]. Standardized evaluation criteria, including histological, radiological, and biomechanical endpoints, further enhance the model's translational value.

A key challenge of the mandible defect model used in this study – and also in clinical setting - lays in the anatomical conditions, which made sufficient plastic coverage of the intraoral area difficult. Since the dimensions of the implants were modeled on physiological bone, significant mobilization of the mucosa and musculature was required to enable tension-free wound adaptation. In the event of suture dehiscence, renewed coverage proved particularly difficult due to the limited availability of soft tissue. If bacterially contaminated saliva or foreign bodies such as food residues or straw entered the pore structure of the implant, the resulting infection could not be adequately controlled by the immune system.

Intraoral dehiscences represented the primary clinical complication in this study, leading to impaired bone regeneration due to infection and inflammatory processes. This finding highlights the critical importance of precise implant adaptation to the individual anatomical situation, raising the question of the degree of customization required for patient-specific implants to optimize regenerative outcomes. A patient-specific implant is generally designed to precisely mirror the anatomical shape and contour of the defect or bone structure to provide an optimal contact surface for primary stability and to minimize micro-movements between the implant and bone, which is critical for osseointegration. Nevertheless, processes such as bone remodeling, scar tissue development, soft tissue atrophy, and alterations in the overlying skin may occur between the time of preoperative planning and the actual surgical procedure. Consequently, it would be advantageous to use an implant that allows for intraoperative adjustment to the defect site and also adapts to some extent as the regeneration process continues. This could potentially be achieved by integrating a buffering layer consisting of a flexible/elastic biomaterial, capable of bridging minor gaps while supporting cellular migration, proliferation, and neovascularization.

Bone regeneration is a multi-phase process (inflammation, repair, remodeling) that extends over months to years in clinically relevant settings. Thus, long-term *in vivo* studies are essential for clinical translation in bone regeneration, as they capture the full interplay between bone remodeling, biomaterial degradation, and mechanical loading under conditions that closely resemble human physiology. Short-term studies (e.g., ≤12 weeks) typically capture only early osteogenesis, but usually not bone maturation, late-onset complications, long-term remodeling and load adaption as well as degradation-associated inflammatory responses and late-stage resorption of biomaterials. In our study this is mirrored by the progressive bone regeneration and implant degradation (PDLLA/SrHAp-C) as well as by the late occurrence of complications (PDLLA, animal #4: fistula at 8 months) observed during the long-term observation period of 14 months. Consequently, such studies are crucial to bridge the gap between proof-of-concept and clinical reality and to reveal time-dependent failure modes that determine clinical success.

### 4.3 Limitations

Several limitations must be considered when interpreting the present findings. First, the exploratory study design and small group size limited the statistical power and precluded definitive conclusions regarding differences among the implant groups. Second, the mechanical properties of the three implant architectures and their evolution during degradation were not experimentally quantified. In addition, total and interconnected porosity could not be quantified retrospectively for all implant components because complete pore-resolved CAD data and as-manufactured volumetric measurements were not available. Consequently, only the reported strand and pore dimensions can be used to describe the scaffold architecture, and comparisons of architecture-dependent effects, particularly for the biphasic SrHAp core, should be interpreted with caution.

Furthermore, preservation of the periosteum during surgery in the present study may have supported bone healing, thereby limiting the direct applicability of the results to clinical situations where periosteal integrity is often compromised. In cases of benign tumors (such as ameloblastoma) and bone cysts, the animal model provides a close approximation to clinical conditions. Potential applications include the regeneration of bone defects in otherwise healthy, young patients with uncompromised bone quality, thereby obviating the need for microvascular grafts (e.g., fibula flaps) or free bone grafts (e.g., iliac crest cancellous or corticocancellous bone), and consequently reducing donor site morbidity associated with graft harvesting. In the case of malignant tumors, the model still deviates significantly from clinical reality. In clinical practice, resection of the periosteum is absolutely necessary, which was not taken into account in the present study. In addition, adjuvant radiation therapy is usually performed in clinics, which causes significant damage to the surrounding soft tissue and bone. This aspect has also not yet been incorporated into the model. It should also be noted that due to the preservation of the periosteum, the approximately 3 cm segmental defect cannot be classified as critical size defect. As demonstrated by Ma et al. [81], the critical size for segmental defects in the mandible of minipigs was determined to be 6 cm with preserved periosteum and 2 cm without periosteum. This is underlined by the fact that complete defect bridging occurred in the present study even when the implant was removed prematurely (PDLLA + SrHAp-P: animal #5).

Furthermore, it should be emphasized that this study utilized only healthy, male minipigs, which not only omits the influence of comorbidities commonly present in human patients, but also precludes the assessment of possible gender-specific differences in bone regeneration. Additionally, the minipigs are also characterized by a high capacity for spontaneous regeneration and re-osseointegration [87] which is in contrast to clinical scenarios that typically involve multimorbid, elderly patients who often present a compromised general health status and diminished regenerative potential, which may be further impaired by factors such as medication or prior radiotherapy.

Finally, the absence of early observation points for histological analyzes precludes detailed insights into the initial stages of bone regeneration, making it difficult to elucidate the temporal dynamics of the healing process. Consequently, while the study presented here is well-suited for drawing important conclusions regarding implant optimization, defect model and surgical procedure, the translational relevance of the findings is limited.

## 5 Summary and outlook

This study assessed the performance of individually manufactured biodegradable implants based on PDLLA in a minipig mandible defect model over a period of up to 14 months. To mitigate the acidic degradation of PDLLA and enhance bone regeneration, strontium-modified hydroxyapatite (SrHAp) was incorporated either by mixing SrHAp microparticles into the PDLLA matrix (PDLLA + SrHAp-P) or by fabricating biphasic PDLLA/SrHAp-C implants with a shell/core architecture.

Across all groups, the high incidence of intraoral dehiscence emphasized the critical importance of soft-tissue management and implant geometry. The large implant dimensions, limited soft-tissue coverage, and restricted capacity to tolerate dimensional changes in the mandibular environment likely contributed to mechanical irritation and wound breakdown. Radiographic defect bridging was observed in all animals by 6 months; however, complete filling of the defect region was not achieved in any animal. Bone formation originated predominantly from the periosteum and along the reconstruction plate rather than through extensive implant-guided ingrowth, indicating that endogenous periosteal regeneration made a major contribution to defect bridging.

A central finding of the study was the mismatch between degradation behavior, mechanical stability, and biological response. Pure PDLLA implants were associated with degradation-related complications, including abscess and fistula formation, consistent with previous reports linking bulk polylactide degradation to acidic degradation products, local pH reduction, and foreign-body reactions. In the present study, these effects may have been intensified by the comparatively large implant volume and the mechanically demanding mandibular environment. Incorporation of SrHAp microparticles into the PDLLA matrix did not overcome these limitations. The PDLLA + SrHAp-P implants exhibited pronounced swelling, loss of dimensional stability, and persistent inflammatory complications, which compromised their space-maintaining function. Although strontium-containing calcium phosphates have been reported to support osteogenic activity, these potential benefits appear to have been outweighed in the present configuration by unfavorable degradation behavior, insufficient architectural stability, and limited accessibility of the ceramic phase.

Among the three implant variants, the biphasic PDLLA/SrHAp-C construct showed the most promising local osteoconductive response, as indicated by the highest measured mineralized-tissue volume within the defect-region volume of interest and by bone ingrowth into the SrHAp core in selected animals. These findings should nevertheless be interpreted cautiously because of the exploratory sample size, the high complication rate, and the inclusion of periosteal and exostotic bone within the radiographic volume of interest. Moreover, the potential biological benefit of the ceramic core was offset by mechanical instability and soft-tissue complications, including core fracture, displacement, and frequent wound dehiscence. The persistence of the SrHAp core may also have limited tissue remodeling and load transfer, although local degradation, vascularization, and mechanical adaptation were not directly quantified.

From a design perspective, several optimization strategies can be derived. First, replacement of PDLLA with a more biologically compatible and dimensionally stable matrix may be more promising than further incremental optimization of the current polymer formulation. Potential alternatives include degradable synthetic polymers with lower acidification and reduced swelling. In addition, predominantly ceramic-based constructs combined with natural polymers, such as collagen or mineralized collagen, may provide a more biomimetic approach by reproducing key features of the organic–inorganic composition of native bone while reducing the burden of acid-generating synthetic polymer. Second, incorporation of a more rapidly resorbing or pore-forming phase into the SrHAp component, such as mesoporous bioactive glass, may increase porosity during degradation and thereby facilitate vascularization, material resorption, and bone ingrowth *in vivo* [88]. Third, the biphasic shell/core architecture should be reconsidered in favor of mechanically better integrated homogeneous, interpenetrating, or functionally graded designs that reduce interfacial stress and improve structural coherence. Fourth, integration of bioactive molecules or pro-angiogenic factors may further support vascularization and bone formation. Richter et al. [30], for example, reported increased bone formation after incorporation of mesoporous bioactive glass loaded with a hypoxia-conditioned medium-derived protein mixture into 3D-printed strontium-containing calcium phosphate cement implants in a rat alveolar cleft model. Finally, improved load-sharing between the implant and osteosynthesis system, together with a reconstruction-plate design of reduced prominence and optimized surgical techniques that minimize soft-tissue tension, will be essential for improving clinical performance.

Taken together, the present implant designs are not yet suitable for clinical translation. Regardless of the selected material system, the most urgent priorities are to ensure stable mechanical integration between the ceramic and matrix phases, preserve interconnected porosity during manufacture and degradation, and reduce the prominence of both the implant and reconstruction plate beneath the oral soft tissues. Soft-tissue complications may be mitigated by rounded and recessed implant contours, tension-free vascularized coverage, minimization of dead space, and a fixation strategy that limits micromotion without creating bulky submucosal components. Although the present study cannot definitively distinguish scaffold-related from plate-related causes, the frequent plate exposure and bone formation around or over the reconstruction plate indicate that the fixation concept should be reconsidered together with the scaffold architecture.

Before first-in-human evaluation, any revised construct should undergo mechanical and fatigue testing before and during degradation, characterization of degradation products and local pH changes, ion-release studies, early histological assessment of inflammation and vascularization, infection-challenge testing, and confirmation in a statistically powered large-animal study incorporating clinically relevant periosteal and soft-tissue compromise. For predominantly ceramic or ceramic–natural polymer systems, additional attention should be paid to handling, fixation, sterilization, structural integrity under cyclic loading, and compatibility with patient-specific additive manufacturing.

The present patient-specific workflow is most compatible with planned oncologic or staged reconstructive procedures rather than immediate emergency reconstruction. Clinical translation will require a validated digital workflow with automated segmentation and design, standardized architecture libraries, parallel manufacture of the reconstruction plate and scaffold, and qualified post-processing and sterilization. In acute trauma, temporary stabilization followed by delayed definitive patient-specific reconstruction, or the use of modular off-the-shelf scaffold components, may be more realistic.

In conclusion, the biphasic configuration showed potentially favorable local osteoconductive properties, but the high complication burden, mechanical instability, and heterogeneous biological response preclude clinical application of the current design. Future translational development should therefore focus not only on architectural, mechanical, and fixation-related optimization, but also on replacement of PDLLA with more biologically compatible and dimensionally stable material systems.

## Declaration of generative AI use

The generative AI tools ChatGPT (OpenAI), You.com, and DeepL were used to assist in identifying recent literature, language editing, improvement of grammar, clarity, and readability, and structuring of the manuscript. All content was critically reviewed, revised, and validated by the authors. No AI tools were used for data analysis, interpretation, or generation of scientific results. The authors take full responsibility for the content of this manuscript.

## Funding sources

The authors thank the German Federal Ministry of Research, Technology and Aerospace (BMFTR) for funding of the initiative ProMatLeben and particularly the subproject “Innovative Polymerkomposite für die patientenindividuelle additive Fertigung biodegradabler Kieferimplantate (InnoPoly)” (grant number 13XP5067).

## CRediT authorship contribution statement

**Christian Bräuer:** Conceptualization, Investigation, Methodology, Writing – original draft, Writing – review & editing. **Elisabeth Jarmer:** Formal analysis, Investigation, Writing – original draft, Writing – review & editing. **Anja Lode:** Conceptualization, Investigation, Project administration, Supervision, Writing – review & editing. **Tatjana Fecht:** Investigation, Methodology, Writing – review & editing. **Stefanie Grom:** Funding acquisition, Methodology, Resources, Writing – review & editing. **Andreas Hoess:** Methodology, Writing – review & editing. **Richard Frank Richter:** Formal analysis, Investigation, Writing – review & editing. **Martin Scharffenberg:** Investigation, Writing – review & editing. **Jakob Wittenstein:** Investigation, Writing – review & editing. **Roland Jung:** Investigation, Writing – review & editing. **Sascha Heinemann:** Funding acquisition, Resources, Writing – review & editing. **Tobias Wolfram:** Conceptualization, Resources, Writing – review & editing. **Frank Reinauer:** Conceptualization, Funding acquisition, Project administration, Resources, Writing – review & editing. **Michael Gelinsky:** Conceptualization, Funding acquisition, Project administration, Writing – review & editing. **Günter Lauer:** Conceptualization, Funding acquisition, Resources, Supervision, Writing – review & editing. **Corina Vater:** Conceptualization, Data curation, Formal analysis, Investigation, Methodology, Supervision, Visualization, Writing – original draft, Writing – review & editing.

## Declaration of competing interest

The authors declare the following financial interests/personal relationships which may be considered as potential competing interests: Andreas Hoess and Sascha Heinemann reports a relationship with INNOTERE GmbH that includes: employment. Tatjana Fecht, Stefanie Grom, Tobias Wolfram, and Frank Reinauer reports a relationship with KLS Martin SE & Co. KG that includes: employment. If there are other authors, they declare that they have no known competing financial interests or personal relationships that could have appeared to influence the work reported in this paper.

## Data Availability

Data will be made available on request.

## References

[1] Cuesta Gil M., Bucci T., Ruiz B.D., Vila C.N., Marenzi G., Sammartino G. (2012). Implant mandibular rehabilitation postoncologic segmental resection: a clinical report. Implant Dent..

[2] Bilkay U., Tokat C., Helvaci E., Ozek C., Alper M. (2004). Free fibula flap mandible reconstruction in benign mandibular lesions. J. Craniofac. Surg..

[3] Kim R., Sokoya M., Ducic Y., Williams F. (2019). Free-flap reconstruction of the mandible. Semin. Plast. Surg..

[4] Molteni G., Gazzini L., Sacchetto A., Nocini R., Comini L.V., Arietti V., Locatello L.G., Mannelli G. (2023). Mandibular reconstruction in head and neck cancer: which is the gold standard?. Eur. Arch. Otorhinolaryngol..

[5] Kumar B.P., Venkatesh V., Kumar K.A.J., Yadav B.Y., Mohan S.R. (2016). Mandibular reconstruction: overview. J. Maxillofac. Oral Surg..

[6] Ling X.F., Peng X., Samman N. (2013). Donor-site morbidity of free fibula and DCIA flaps. J. Oral Maxillofac. Surg..

[7] Ling X.F., Peng X. (2012). What is the price to pay for a free fibula flap? A systematic review of donor-site morbidity following free fibula flap surgery. Plast. Reconstr. Surg..

[8] Tevlin R., McArdle A., Atashroo D., Walmsley G.G., Senarath-Yapa K., Zielins E.R., Paik K.J., Longaker M.T., Wan D.C. (2014). Biomaterials for craniofacial bone engineering. J. Dent. Res..

[9] Klotch D.W., Prein J. (1987). Mandibular reconstruction using AO plates. Am. J. Surg..

[10] Sadr-Eshkevari P., Rashad A., Vahdati S.A., Garajei A., Bohluli B., Maurer P. (2013). Alloplastic mandibular reconstruction: a systematic review and meta-analysis of the current century case series. Plast. Reconstr. Surg..

[11] Kämmerer P.W., Klein M.O., Moergel M., Gemmel M., Draenert G.F. (2014). Local and systemic risk factors influencing the long-term success of angular stable alloplastic reconstruction plates of the mandible. J. Cranio-Maxillofacial Surg..

[12] Ferretti C., Rikhotso E., Muthray E., Reyneke J. (2013). Interim reconstruction and space maintenance of mandibular continuity defects preceding definitive osseous reconstruction. Br. J. Oral Maxillofac. Surg..

[13] Goodger N.M., Wang J., Smagalski G.W., Hepworth B. (2005). Methylmethacrylate as a space maintainer in mandibular reconstruction. J. Oral Maxillofac. Surg..

[14] Henslee A.M., Spicer P.P., Shah S.R., Tatara A.M., Kasper F.K., Mikos A.G., Wong M.E. (2014). Use of porous space maintainers in staged mandibular reconstruction. Oral Maxillofac. Surg. Clin..

[15] Teschke M., Christensen A., Far F., Reich R.H., Naujokat H. (2021). Digitally designed, personalized bone cement spacer for staged TMJ and mandibular reconstruction - introduction of a new technique. J. Craniomaxillofac. Surg..

[16] Bräuer C., Ullmann K., Lauer G., Franke A., McLeod N.M.H., Leonhardt H. (2023). Alloplastic reconstruction of the mandible after subtotal mandibulectomy for medication‐related osteonecrosis of the jaw: an update of the method. Head Neck.

[17] Menchisheva Y., Varela Morillas A., Frascione N. (2025). Polyetheretherketone for craniomaxillofacial defects: cases report, evaluation of patients' satisfaction and a systematic literature review. Maxillofac Plast Reconstr Surg.

[18] Memon A.R., Wang E., Hu J., Egger J., Chen X. (2020). A review on computer-aided design and manufacturing of patient-specific maxillofacial implants. Expert Rev. Med. Dev..

[19] Narayanan G., Vernekar V.N., Kuyinu E.L., Laurencin C.T. (2016). Poly (lactic acid)-based biomaterials for orthopaedic regenerative engineering. Adv. Drug Deliv. Rev..

[20] Annunziata M., Nastri L., Cecoro G., Guida L. (2017). The use of poly-d,l-lactic acid (PDLLA) devices for bone augmentation techniques: a systematic review. Molecules.

[21] Madhavan Nampoothiri K., Nair N.R., John R.P. (2010). An overview of the recent developments in polylactide (PLA) research. Bioresour. Technol..

[22] Grémare A., Guduric V., Bareille R., Heroguez V., Latour S., L’heureux N., Fricain J., Catros S., Le Nihouannen D. (2018). Characterization of printed PLA scaffolds for bone tissue engineering. J. Biomed. Mater. Res..

[23] Ahlfeld T., Lode A., Placht A.-M., Fecht T., Wolfram T., Grom S., Hoess A., Vater C., Bräuer C., Heinemann S., Lauer G., Reinauer F., Gelinsky M. (2023). A comparative analysis of 3D printed scaffolds consisting of poly(lactic- *co* -glycolic) acid and different bioactive mineral fillers: aspects of degradation and cytocompatibility. Biomater. Sci..

[24] Vater C., Bräuer C., Grom S., Fecht T., Ahlfeld T., Von Witzleben M., Placht A.-M., Schütz K., Schehl J.M., Wolfram T., Reinauer F., Scharffenberg M., Wittenstein J., Hoess A., Heinemann S., Gelinsky M., Lauer G., Lode A. (2024). Poly(dl-lactide) polymer blended with mineral phases for extrusion 3D printing—studies on degradation and biocompatibility. Polymers.

[25] Hentschel L., Petersmann S., Gonzalez-Gutierrez J., Kynast F., Schäfer U., Arbeiter F., Holzer C. (2023). Parameter optimization of the ARBURG plastic freeforming process by means of a design of experiments approach. Adv. Eng. Mater..

[26] Liu H., Slamovich E.B., Webster T.J. (2006). Less harmful acidic degradation of poly(lacticco-glycolic acid) bone tissue engineering scaffolds through titania nanoparticle addition. Int. J. Nanomed..

[27] Tsunoda M. (2003). Degradation of poly (DL-Lactic acid-co-glycolic acid) containing calcium carbonate and hydroxyapatite fillers-effect of size and shape of the Fillers-. Dent. Mater. J..

[28] Agrawal C.M., Athanasiou K.A. (1997). Technique to control pH in vicinity of biodegrading PLA-PGA implants. J. Biomed. Mater. Res..

[29] Lode A., Heiss C., Knapp G., Thomas J., Nies B., Gelinsky M., Schumacher M. (2018). Strontium-modified premixed calcium phosphate cements for the therapy of osteoporotic bone defects. Acta Biomater..

[30] Richter R.F., Vater C., Korn M., Ahlfeld T., Rauner M., Pradel W., Stadlinger B., Gelinsky M., Lode A., Korn P. (2023). Treatment of critical bone defects using calcium phosphate cement and mesoporous bioactive glass providing spatiotemporal drug delivery. Bioact. Mater..

[31] Von Witzleben M., Ahlfeld T., Richter R.F., Placht A.-M., Greil C., Vater C., Hoess A., Heinemann S., Grom S., Fecht T., Schehl J.M., Wolfram T., Reinauer F., Bräuer C., Lauer G., Gelinsky M., Lode A. (2026). Biphasic bone implants through hybrid extrusion printing of thermoplastic Poly(lactic- *co* -glycolic) acid and strontium-modified calcium phosphate bone cement. ACS Omega.

[32] Schumacher M., Lode A., Helth A., Gelinsky M. (2013). A novel strontium(II)-modified calcium phosphate bone cement stimulates human-bone-marrow-derived mesenchymal stem cell proliferation and osteogenic differentiation in vitro. Acta Biomater..

[33] Thormann U., Ray S., Sommer U., ElKhassawna T., Rehling T., Hundgeburth M., Henß A., Rohnke M., Janek J., Lips K.S., Heiss C., Schlewitz G., Szalay G., Schumacher M., Gelinsky M., Schnettler R., Alt V. (2013). Bone formation induced by strontium modified calcium phosphate cement in critical-size metaphyseal fracture defects in ovariectomized rats. Biomaterials.

[34] Percie Du Sert N., Hurst V., Ahluwalia A., Alam S., Avey M.T., Baker M., Browne W.J., Clark A., Cuthill I.C., Dirnagl U., Emerson M., Garner P., Holgate S.T., Howells D.W., Karp N.A., Lazic S.E., Lidster K., MacCallum C.J., Macleod M., Pearl E.J., Petersen O.H., Rawle F., Reynolds P., Rooney K., Sena E.S., Silberberg S.D., Steckler T., Würbel H. (2020). The ARRIVE guidelines 2.0: updated guidelines for reporting animal research. PLoS Biol..

[35] Panes C., Ponce N., Ottone N.E., Valdivia-Gandur I., Beltrán V., Vásquez B. (2024). Modified goldner trichrome for non-decalcified mineralized tissue plastinated and embedded in resin. Int. J. Morphol..

[36] Guo W., Li P., Wei Y., Zhao L., Pang Y., Huang Y., Ye X., Wang S., Liu B., You H., Long Y. (2024). Ionic substitution through bredigite doping for microstructure and performance adjustment in DLP 3D-printed TPMS porous HA bone scaffolds. Virtual Phys. Prototyp..

[37] Guo W., Li P., Pang Y., Wang E., Zhao L., Huang Y., Wang S., Liu B., You H., Long Y. (2025). Biomimetic TPMS porous hydroxyapatite bone scaffolds doped with bioactive glass: digital light processing additive manufacturing, microstructure and performance. Compos. Appl. Sci. Manuf..

[38] Guo W., Feng S., Li B., Xu C., Wei Y., Gong Y., Wang S., Mai H., Zhang Y., Long Y. (2025). Niobium carbide-functionalized polylactic acid bone scaffold with near-infrared light-mediated shape memory and photothermal therapy for complex bone defects. Int. J. Biol. Macromol..

[39] Guo W., Wei Y., Xu C., Li B., Wu Y., Gong Y., Mai H., Wang S., Zhang Y., Long Y. (2026). Functionalized degradable soybean oil-based biomimetic porous scaffolds for complex bone defects: vat photopolymerization additive manufacturing, photothermal-mediated shape memory and tumor thermotherapy. Compos. Sci. Technol..

[40] Guo W., Tang X., Li B., Xu C., Huang Y., Wang S., Zhong Y., Gong M., Ding K., Long Y. (2026). Multi-element CuFeSe2 nanoparticle-functionalized 3D-printed polylactic acid scaffolds with photothermal shape memory and therapy for complex bone defects. Ceram. Int..

[41] Heidemann W., Jeschkeit S., Ruffieux K., Fischer J.H., Wagner M., Krüger G., Wintermantel E., Gerlach K.L. (2001). Degradation of poly(d, l)lactide implants with or without addition of calciumphosphates in vivo. Biomaterials.

[42] Xue A.S., Koshy J.C., Weathers W.M., Wolfswinkel E.M., Kaufman Y., Sharabi S.E., Brown R.H., Hicks M.J., Hollier L.H. (2014). Local foreign-body reaction to commercial biodegradable implants: an in vivo animal study. Craniomaxillofacial Trauma Reconstr..

[43] Nonhoff M., Puetzler J., Hasselmann J., Fobker M., Gosheger G., Schulze M. (2024). The potential for foreign body reaction of implanted Poly-L-Lactic acid: a systematic review. Polymers.

[44] Yi J., Xiong F., Li B., Chen H., Yin Y., Dai H., Li S. (2016). Degradation characteristics, cell viability and host tissue responses of PDLLA-based scaffold with PRGD and β-TCP nanoparticles incorporation. Regen. Biomater..

[45] Turvey T.A., Proffit W.P., Phillips C. (2011). Biodegradable fixation for craniomaxillofacial surgery: a 10-year experience involving 761 operations and 745 patients. Int. J. Oral Maxillofac. Surg..

[46] Pina S., Ferreira J.M.F. (2012). Bioresorbable plates and screws for clinical applications: a review. J. Healthc. Eng..

[47] Landes C.A., Ballon A., Roth C. (2006). Maxillary and mandibular osteosyntheses with PLGA and P(L/DL)LA implants: a 5-Year inpatient biocompatibility and degradation experience. Plast. Reconstr. Surg..

[48] Blaker J.J., Nazhat S.N., Maquet V., Boccaccini A.R. (2011). Long-term in vitro degradation of PDLLA/Bioglass® bone scaffolds in acellular simulated body fluid. Acta Biomater..

[49] Feng P., Jia J., Liu M., Peng S., Zhao Z., Shuai C. (2021). Degradation mechanisms and acceleration strategies of poly (lactic acid) scaffold for bone regeneration. Mater. Des..

[50] Li R.-Y., Liu Z.-G., Liu H.-Q., Chen L., Liu J.-F., Pan Y.-H. (2015). Evaluation of biocompatibility and toxicity of biodegradable poly (DL-lactic acid) films. Am. J. Transl. Res..

[51] Scorsato P.S., Rahal S.C., Cestari T.M., Mamprim M.J., Doiche D.P., Teixeira D. de B., Siqueira R.C., Felix M. (2022). Evaluation of the degradation of two bioabsorbable interference screws: an in-vivo study in sheep. Acta Cir. Bras..

[52] Heidemann W., Fischer J.H., Koebke J., Bussmann C., Gerlach K.L. (2003). In-vivo-Untersuchung zur Degradation von Poly-(D,L-)Laktid- und Poly-(L-Laktid-co-Glykolid)-Osteosynthesematerial. Mund-, Kiefer- Gesichtschirurgie.

[53] Taş C., Kozluca A., Onur M.A., Tümer A., Vahapoğlu H., Zareie M.H., Gunning P.A., Pişkin E. (1998). In vivo degradation and release kinetics of chloramphenicol-loaded poly(D,L)-lactide sponges. Tissue Eng..

[54] Wu L., Ding J. (2004). In vitro degradation of three-dimensional porous poly(d,l-lactide-co-glycolide) scaffolds for tissue engineering. Biomaterials.

[55] Pilling E., Mai R., Theissig F., Stadlinger B., Loukota R., Eckelt U. (2007). An experimental in vivo analysis of the resorption to ultrasound activated pins (sonic weld®) and standard biodegradable screws (ResorbX®) in sheep. Br. J. Oral Maxillofac. Surg..

[56] Shikinami Y., Okazaki K., Saito M., Okuno M., Hasegawa S., Tamura J., Fujibayashi S., Nakamura T. (2006). Bioactive and bioresorbable cellular cubic-composite scaffolds for use in bone reconstruction. J. R. Soc., Interface.

[57] Heidemann W., Jeschkeit-Schubbert S., Ruffieux K., Fischer J.H., Jung H., Krueger G., Wintermantel E., Gerlach K.L. (2002). pH-stabilization of predegraded PDLLA by an admixture of water-soluble sodiumhydrogenphosphate—results of an in vitro- and in vivo-study. Biomaterials.

[58] Nkenke E., Vairaktaris E., Knipfer C., Stelzle F., Schwarz S., Eyüpoglu I., Ganslandt O., Leis T. (2011). Prospective assessment of complications associated with ultrasound activated resorbable pin osteosynthesis in pediatric craniofacial surgery: preliminary results. Neurocirugía.

[59] Rasse M., Moser D., Zahl C., Gerlach K.L., Eckelt U., Loukota R. (2007). Resorbable poly(d, l)lactide plates and screws for osteosynthesis of condylar neck fractures in sheep. Br. J. Oral Maxillofac. Surg..

[60] Duong L.T., Petit S., Kerner S., Clerc M.M., Arnoult C., Nowwarote N., Osathanon T., Fournier B.P.J., Isaac J., Ferré F.C. (2023). Role of periosteum during healing of alveolar critical size bone defects in the mandible: a pilot study. Clin. Oral Invest..

[61] Shanbhag S., Kampleitner C., Sanz‐Esporrin J., Lie S., Gruber R., Mustafa K., Sanz M. (2024). Regeneration of alveolar bone defects in the experimental pig model: a systematic review and meta‐analysis. Clin. Oral Implants Res..

[62] Sillmann Y.M., Eber P., Orbeta E., Wilde F., Gross A.J., Guastaldi F.P.S. (2025). Milestones in mandibular bone tissue engineering: a systematic review of large animal models and critical-sized defects. JCM.

[63] Probst F.A., Fliefel R., Burian E., Probst M., Eddicks M., Cornelsen M., Riedl C., Seitz H., Aszódi A., Schieker M., Otto S. (2020). Bone regeneration of minipig mandibular defect by adipose derived mesenchymal stem cells seeded tri-calcium phosphate- poly(D,L-lactide-co-glycolide) scaffolds. Sci. Rep..

[64] Bouyer M., Garot C., Machillot P., Vollaire J., Fitzpatrick V., Morand S., Boutonnat J., Josserand V., Bettega G., Picart C. (2021). 3D-printed scaffold combined to 2D osteoinductive coatings to repair a critical-size mandibular bone defect. Mater. Today Bio.

[65] Raghoebar G.M., Liem R.S.B., Bos R.R.M., van der Wal J.E., Vissink A. (2006). Resorbable screws for fixation of autologous bone grafts. Clin. Oral Implants Res..

[66] Achtnich A., Forkel P., Metzlaff S., Zantop T., Petersen W. (2014). Degradation of poly-D-L-lactide (PDLLA) interference screws (Megafix ®). Arch. Orthop. Trauma Surg..

[67] Ho-Shui-Ling A., Bolander J., Rustom L.E., Johnson A.W., Luyten F.P., Picart C. (2018). Bone regeneration strategies: engineered scaffolds, bioactive molecules and stem cells current stage and future perspectives. Biomaterials.

[68] Furukawa T., Matsusue Y., Yasunaga T., Shikinami Y., Okuno M., Nakamura T. (2000). Biodegradation behavior of ultra-high-strength hydroxyapatite/poly (L-lactide) composite rods for internal fixation of bone fractures. Biomaterials.

[69] Wang Z., Wang Y., Ito Y., Zhang P., Chen X. (2016). A comparative study on the in vivo degradation of poly(L-lactide) based composite implants for bone fracture fixation. Sci. Rep..

[70] Xiao Y.M., Fan H.S., Wang X.L., Xu J.R., Cheng J.Q., Li X.D., Zhang X.D. (2005). Preparation of Nano-Hydroxyapatite/Poly(D,L-Lactide) composite and its degradation in femora of rabbits. KEM.

[71] Dong Q.N., Kanno T., Bai Y., Sha J., Hideshima K. (2019). Bone regeneration potential of uncalcined and unsintered hydroxyapatite/poly l-lactide bioactive/osteoconductive sheet used for maxillofacial reconstructive surgery: an in vivo study. Materials.

[72] Lv S., Liu X., Sui J., Bai C., Fan B., Zhang W., Yuan P., Zhu J., Li J., Shao B. (2024). In vivo research on 3D-printed composite PLGA and PDLLA-HA absorbable scaffolds for repairing radius defects in rabbits. J. Int. Med. Res..

[73] Kuang G.-M., Yau W.P., Wu J., Yeung K.W.K., Pan H., Lam W.M., Lu W.W., Chiu K.Y. (2015). Strontium exerts dual effects on calcium phosphate cement: accelerating the degradation and enhancing the osteoconductivity both in vitro and in vivo. J. Biomed. Mater. Res..

[74] Schröter L., Kaiser F., Stein S., Gbureck U., Ignatius A. (2020). Biological and mechanical performance and degradation characteristics of calcium phosphate cements in large animals and humans. Acta Biomater..

[75] Richter R.F., Pries F., Alt V., Lode A., Heinemann S. (2025). Application of 3D printed calcium phosphate cement scaffolds in open wedge high tibial osteotomy (owHTO) – a retrospective clinical evaluation. Materialia.

[76] Zhao R., Yang R., Cooper P.R., Khurshid Z., Shavandi A., Ratnayake J. (2021). Bone grafts and substitutes in dentistry: a review of current trends and developments. Molecules.

[77] Yang F., Yang D., Tu J., Zheng Q., Cai L., Wang L. (2011). Strontium enhances osteogenic differentiation of mesenchymal stem cells and in vivo bone formation by activating Wnt/catenin signaling. Stem Cell..

[78] Schumacher M., Wagner A.S., Kokesch-Himmelreich J., Bernhardt A., Rohnke M., Wenisch S., Gelinsky M. (2016). Strontium substitution in apatitic CaP cements effectively attenuates osteoclastic resorption but does not inhibit osteoclastogenesis. Acta Biomater..

[79] Korn P., Ahlfeld T., Lahmeyer F., Kilian D., Sembdner P., Stelzer R., Pradel W., Franke A., Rauner M., Range U., Stadlinger B., Lode A., Lauer G., Gelinsky M. (2020). 3D printing of bone grafts for cleft alveolar osteoplasty – in vivo evaluation in a preclinical model. Front. Bioeng. Biotechnol..

[80] Wu R., Li Y., Shen M., Yang X., Zhang L., Ke X., Yang G., Gao C., Gou Z., Xu S. (2021). Bone tissue regeneration: the role of finely tuned pore architecture of bioactive scaffolds before clinical translation. Bioact. Mater..

[81] Ma J.-L., Pan J.-L., Tan B.-S., Cui F.-Z. (2009). Determination of critical size defect of minipig mandible. J. Tissue Eng. Regen. Med..

[82] Lemperle S.M., Calhoun C.J., Curran R.W., Holmes R.E. (1998). Bony healing of large cranial and mandibular defects protected from soft-tissue interposition: a comparative study of spontaneous bone regeneration, osteoconduction, and cancellous autografting in dogs. Plast. Reconstr. Surg..

[83] Kauffmann P., Raschke D., Tröltzsch M., Santander P., Brockmeyer P., Schliephake H. (2021). The use of rhBMP2 for augmentation of established horizontal/vertical defects may require additional use of rhVEGF to achieve significant bone regeneration: an in vivo experimental study. Clin. Oral Implants Res..

[84] Mountziaris P.M., Mikos A.G. (2008). Modulation of the inflammatory response for enhanced bone tissue regeneration. Tissue Eng. B Rev..

[85] Schmidt-Bleek K., Schell H., Schulz N., Hoff P., Perka C., Buttgereit F., Volk H.-D., Lienau J., Duda G.N. (2012). Inflammatory phase of bone healing initiates the regenerative healing cascade. Cell Tissue Res..

[86] van Oirschot B.A.J.A., Geven E.J.W., Mikos A.G., van den Beucken J.J.J.P., Jansen J.A. (2022). A mini-pig mandibular defect model for evaluation of craniomaxillofacial bone regeneration. Tissue Eng. C Methods.

[87] Shanbhag S., Sanz-Esporrin J., Kampleitner C., Lie S.-A., Gruber R., Mustafa K., Sanz M. (2024). Peri-implant bone regeneration in pigs. Int. J. Implant Dent..

[88] Ray S., Thormann U., Kramer I., Sommer U., Budak M., Schumacher M., Bernhardt A., Lode A., Kern C., Rohnke M., Heiss C., Lips K.S., Gelinsky M., Alt V. (2023). Mesoporous bioactive glass-incorporated injectable strontium-containing calcium phosphate cement enhanced osteoconductivity in a critical-sized metaphyseal defect in osteoporotic rats. Bioengineering (Basel).

