# Supplementary material for: Preclinical evaluation of patient-specific 3D-printed PDLLA/SrHAp implants in a minipig segmental mandibular defect model
Source: Mater Today Bio. 2026 Aug 1;40:103523. doi: 10.1016/j.mtbio.2026.103523 (PMC13473966; doi:10.1016/j.mtbio.2026.103523)
Supplement: Supplementary file 1 [file mmc1.docx]

**Supplementary Material**

**Individual implant dimensions**

**Supplemental Figure A1.** Dimensions of the final printed implants of each individual animal (* width and height of the SrHAp core in anterior direction, n. d. – not detectable). Volumes were calculated based on the outer form / the printing STL-files using PrusaSlicer (version 2.9.0).

**Design of the implant variants**

**Supplemental Figure A2.** Design of the 3D-printed porous implants used for implantation: control PDLLA (left), composite PDLLA + SrHAp-P (middle) and biphasic PDLLA / SrHAp-C.

**Design of the reconstruction plate**

**Supplemental Figure A3.** Design of the reconstruction plate used in the study (left: schematic illustration with numbered drilling holes, right: situs of the plate in a porcine cadaver model). For fixation of the plate to the bone, titanium screws at different diameters and length were used depending on the individual anatomic situation (drilling holes at position 1–5: Ø 2.3 mm, length: 7 mm; drilling holes at position 6–13: Ø 2.7 mm, length: 11–19 mm).

**Evaluation of newly formed bone volume**

**Supplemental Figure A4.** Methodology for evaluation of newly formed bone based on DVT data. (A) Volume of interest (VOI) with corresponding slice planes and (B) comparison of the recorded bone volume depending on the density measurement range in the defect area (yellow). All density values above 705 mg/cm³ were defined as osteosynthesis material (blue).

**Weight development**

**Supplemental Figure A5.** Weight development of the minipigs during the 6-months observation period (mean ± SD, n = 4 animals per group, 1-way ANOVA followed by Tukey’s multiple-comparisons test).

**Complications**

| **implant type** | **animal** | **adverse event & time of onset** | **management** | **outcome** |
| --- | --- | --- | --- | --- |
| PDLLA | #1 | extraoral abscess @ 8W  extraoral fistula @ 9W | abscess drainage + antiseptic irrigation; systemic antibiotics + analgesia | defect bridging |
|  | #2 | small intraoral dehiscence @ 5W | none |  |
|  | #3 | intraoral dehiscence @ 5W  extraoral abscess @ 5M  reconstruction plate loosening @ 6M | abscess drainage + antiseptic irrigation; systemic antibiotics + analgesia |  |
|  | #4* | extraoral fistula @ 9M | none |  |
| PDLLA + SrHAp-P | #5 | large intraoral dehiscence @ 5W  implant swelling @ 2M | revision surgery to close dehiscence; implant removal; systemic antibiotics + analgesia |  |
|  | #6 | small intraoral dehiscence @ 5W | none |  |
|  | #7 | intraoral dehiscence @ 4M | none |  |
|  | #8* | very small intraoral dehiscence @ 4M | none |  |
| PDLLA / SrHAp-C | #9 | small intraoral fistula @ 5W  extraoral abscess @ 6W  implant dislocation @ 4M | abscess drainage + antiseptic irrigation |  |
|  | #10 | extraoral fistula @ 2M  small intraoral dehiscence @ 6M | none |  |
|  | #11 | small intraoral dehiscence @ 5W  implant dislocation @ 4M | none |  |
|  | #12* | none | not applicable |  |

* animals assigned to 14-months follow-up without terminal sacrifice

**Supplemental Table S1.** Detailed animal-level summary of complication type, time of onset, treatment, and outcome during the postoperative observation period.

**Qualitative analysis of bone formation over 6 months**

**Supplemental Figure A6.** Bone regeneration in animals of the PDLLA group as evaluated by DVT measurements over time in transverse (red) and coronal (cyan) view as well as DVT-based 3D reconstructions at 6 months postoperatively. The density color overlay is provided as a qualitative aid for distinguishing structures with different radiopacity and should not be interpreted as a calibrated quantitative density measurement. Images were selected to illustrate changes in implant appearance, position, and surrounding bone formation over time. Images within the same anatomical view were displayed using comparable orientation and field of view (DVT voxel size = 400 µm).

**Supplemental Figure A7.** Bone regeneration in animals of the PDLLA + SrHAp-P group as evaluated by DVT measurements over time in transversal (red) and coronar (cyan) view as well as DVT-based 3D reconstructions at 6 months postoperatively. The density color overlay is provided as a qualitative aid for distinguishing structures with different radiopacity and should not be interpreted as a calibrated quantitative density measurement. Images were selected to illustrate changes in implant appearance, position, and surrounding bone formation over time. Images within the same anatomical view were displayed using comparable orientation and field of view (DVT voxel size = 400 µm).

**Supplemental Figure A8.** Bone regeneration in animals of the PDLLA / SrHAp-C group as evaluated by DVT measurements over time in transversal (red) and coronar (cyan) view as well as DVT-based 3D reconstructions at 6 months postoperatively. The density color overlay is provided as a qualitative aid for distinguishing structures with different radiopacity and should not be interpreted as a calibrated quantitative density measurement. Images were selected to illustrate changes in implant appearance, position, and surrounding bone formation over time. Images within the same anatomical view were displayed using comparable orientation and field of view (DVT voxel size = 400 µm).

**Supplemental Figure A9.** Dislocated but osseointegrated SrHAp core (yellow triangles) of animal #11 in transverse (red), coronal (cyan), and sagittal (green) view.

**Evaluation of bone formation using fluorescent polychrome labeling**

**Supplemental Figure A10.** Specific observations in the context of fluorochrome staining. (A) Width of the color bands and direction of new bone formation (animal #8, PDLLA + SrHAp-P) and (B) inhibited growth front originating from the resection margins/bone stumps (animal #3, PDLLA; white dashed lines: resection margins, orange dashed line and orange arrows: gap between formed bone and reconstruction plate, white arrows: direction of new bone formation, blue triangles: osteosynthesis material).

**Detailed histological analysis**

**Supplemental Figure A11.** Masson-Goldner staining. (A) Non-mineralized fibrous tissue at the growth fronts and around the reconstruction plate (animal #1 – PDLLA; left: longitudinal section, right: cross section; dashed white line: defect area, yellow asterisks: fibrous tissue, blue triangles: reconstruction plate). (B) Osseointegrated (green hook) and non-osseointegrated (red cross) osteosynthesis screws (animal #6 – PDLLA + SrHAp-P; blue triangles: osteosynthesis screws).

**Supplemental Figure A12.** Trichrome staining for elastic fibers (TEF-staining) for histological representation of different stages of bone development (marked with yellow triangles, derived from different animals). (A) chondral ossification, (B) immature cancellous bone, (C) formation of osteons, (D) increased trabecular formation, (E) bone structure of the operated side, and (F) bone structure of the contralateral side (yellow asterisks: medullary cavities). The lingual half of the explanted specimens was used for detailed examinations with various histological stains. Therefore, bone samples were stored for 3 weeks in ethylenediaminetetraacetic acid (EDTA) for decalcification. After dehydration overnight, samples were embedded in paraffin and sectioned with a thickness of 2 µm.

**Supplemental Figure A13.** Histological representation of conspicuities in the defect area (from different animals). (A) Cartilaginous areas (Herovici staining, yellow triangles), (B)–(E) Trichrome staining for elastic fibers (TEF-staining) with (B) transition from old to newly formed bone (dashed yellow line), (C) fistula and (D, E) periosteum of the (D) healthy and (E) operated side with thickness of the stratum osteogenicum (yellow triangles). The lingual half of the explanted specimens was used for detailed examinations with various histological stains. Therefore, bone samples were stored for 3 weeks in ethylenediaminetetraacetic acid (EDTA) for decalcification. After dehydration overnight, samples were embedded in paraffin and sectioned with a thickness of 2 µm.

**Premature implant removal (PDLLA + SrHAp-P)**

**Supplemental Figure A14.** Macroscopic image of the PDLLA + SrHAp-P implant (animal #5) removed prematurely 14 weeks after surgery due to swelling and soft tissue dehiscence.
